# Supplementary material for: CALPHAD accelerated design of advanced full-Zintl thermoelectric device
Source: Nat Commun. 2024 Feb 17;15:1468. doi: 10.1038/s41467-024-45869-w (PMC11258146; doi:10.1038/s41467-024-45869-w)
Supplement: Supplementary file 1 — Supplementary Information [file 41467_2024_45869_MOESM1_ESM.pdf]

Supplementary Materials for

CALPHAD accelerated design of advanced full-Zintl thermoelectric device

Li Yin†, Xiaofang Li†, Xin Bao, Jinxuan Cheng, Chen Chen, Zongwei Zhang, Xingjun Liu, Feng Cao, Jun Mao, and Qian Zhang*

†These authors contributed equally to this work.

*Correspondence to: zhangqf@hit.edu.cn

This supplement file includes

Supplementary Figures 1-29
Supplementary Tables 1-9

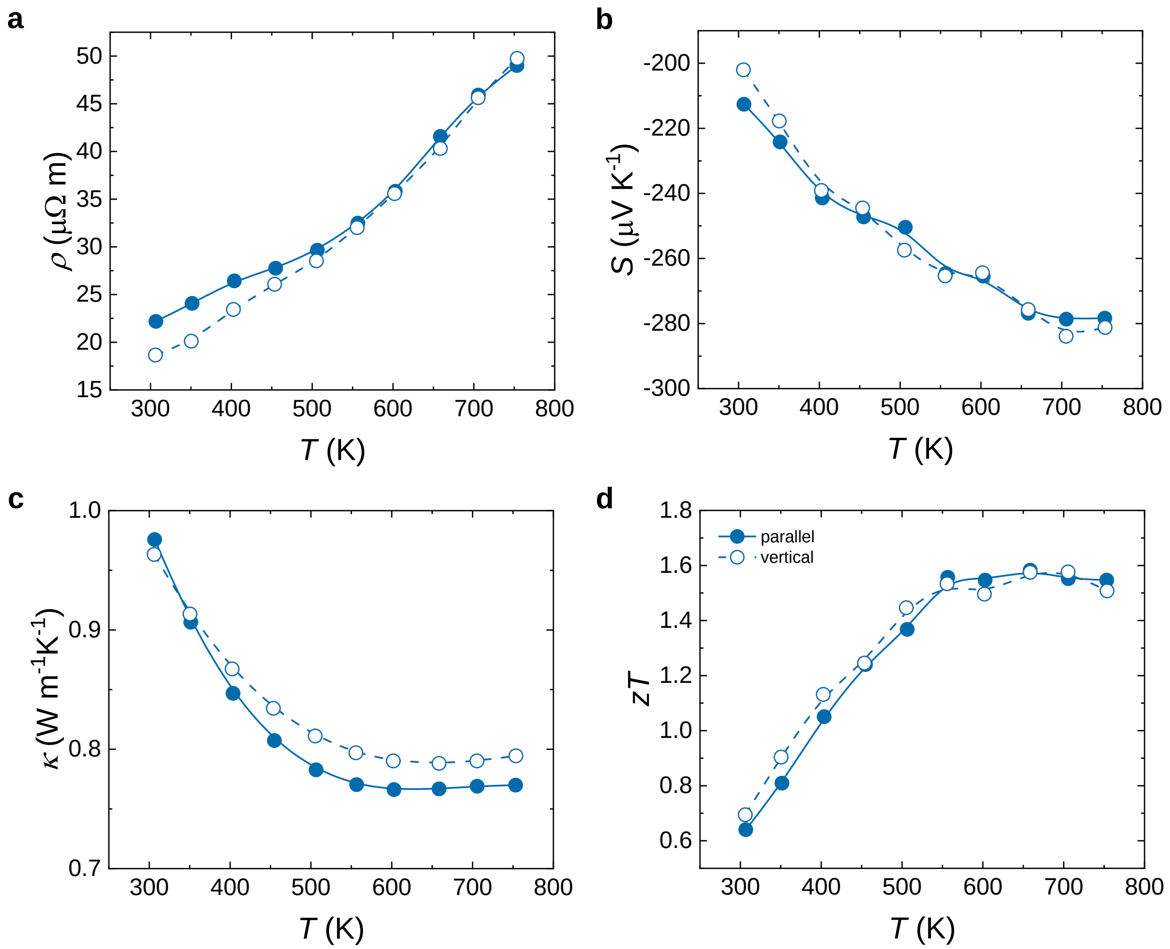

Supplementary Figure 1| Temperature-dependent thermoelectric properties of the n-type $Mg_{3.15}Co_{0.05}SbBi_{0.99}Se_{0.01}$ materials. (a) Electrical resistivity, (b) Seebeck coefficient, (c) thermal conductivity, and (d) *zT* value as a function of temperature.

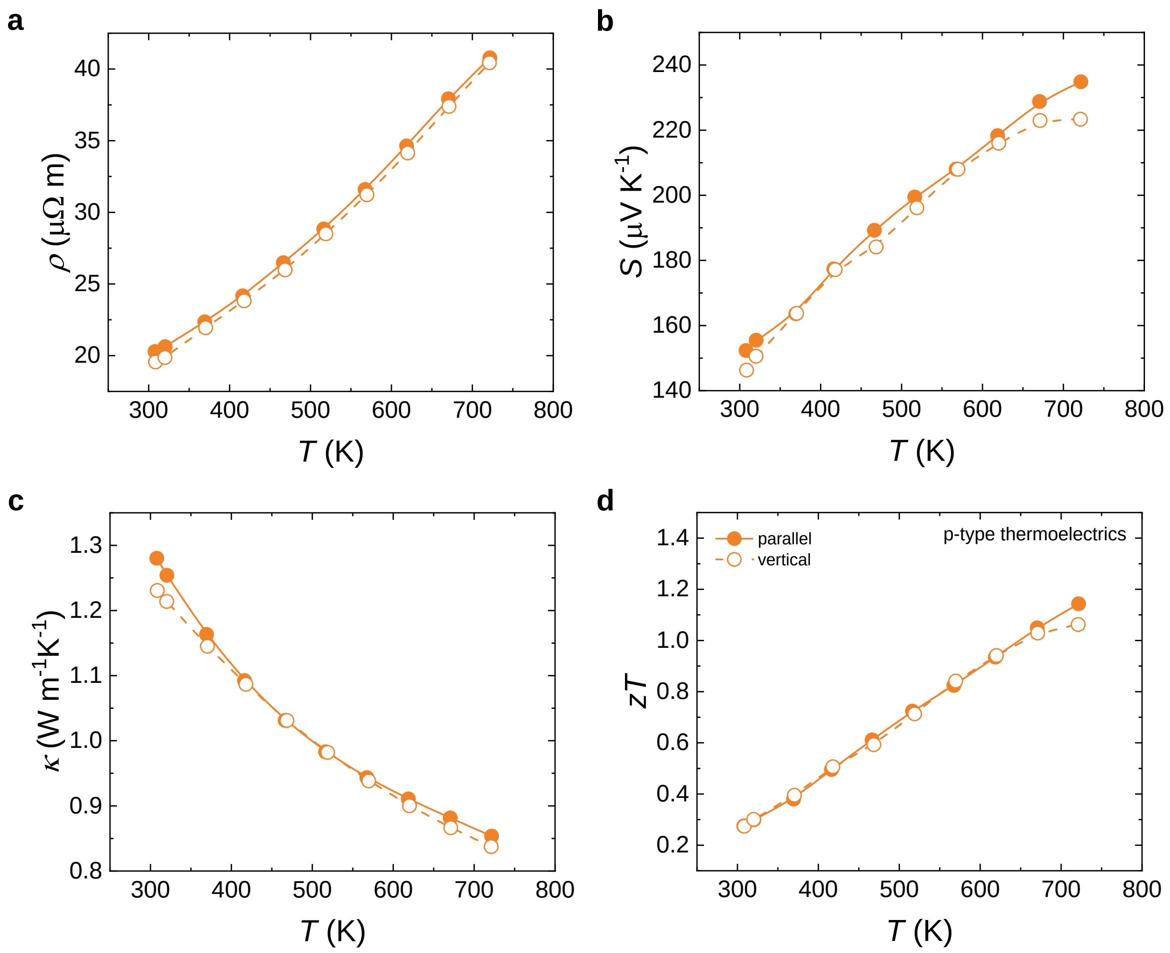

Supplementary Figure 2| Temperature-dependent thermoelectric properties of the p-type $Yb_{0.9}Mg_{0.9}Zn_{1.198}Ag_{0.002}Sb_2$ materials. (a) Electrical resistivity, (b) Seebeck coefficient, (c) thermal conductivity, and (d) *zT* value as a function of temperature.

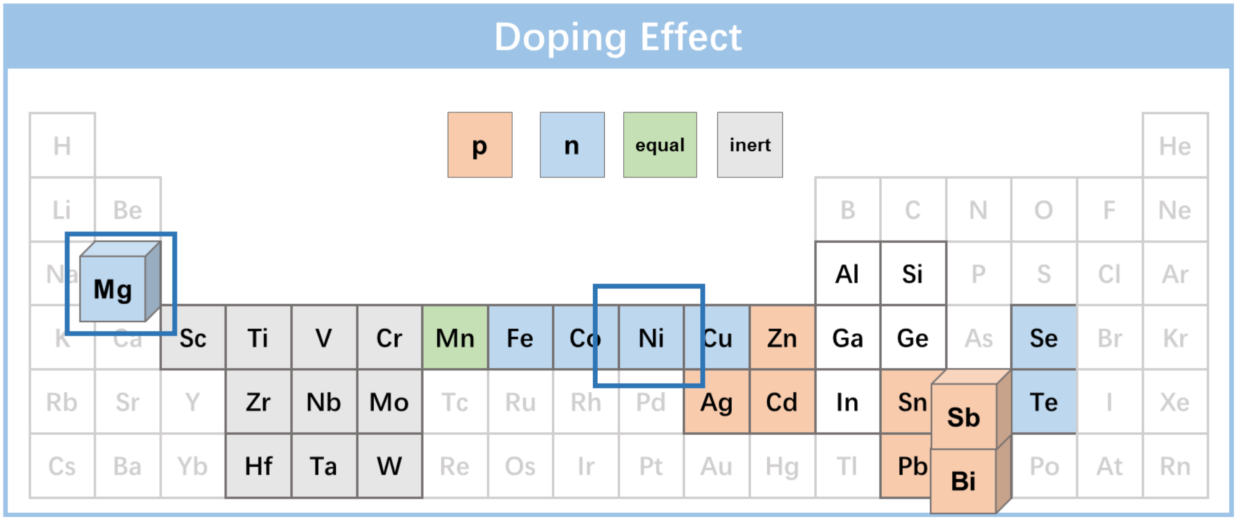

Supplementary Figure 3| The doping effect of elements in Mg_3Sb_2-based materials that has been reported[25-30].

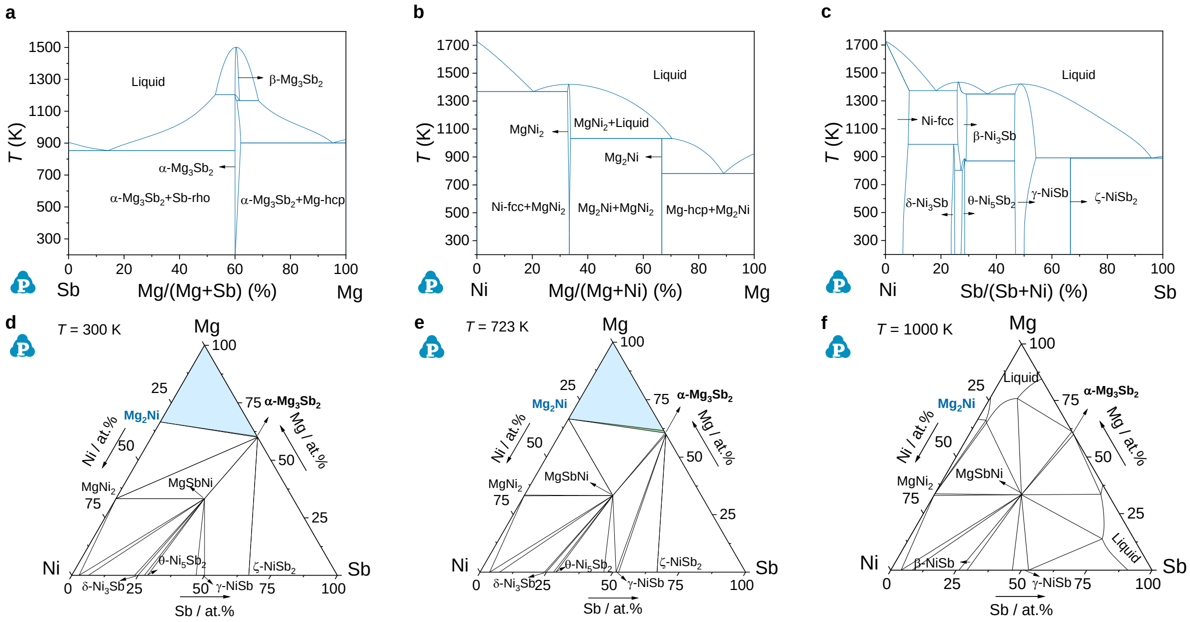

Supplementary Figure 4| The calculated phase diagram. Calculated (a) Mg-Sb binary phase diagram, (b) Mg-Ni binary phase diagram, (c) Ni-Sb binary phase diagram, isothermal sections at (d) 300 K, (c)723 K, and (f) 1000 K.

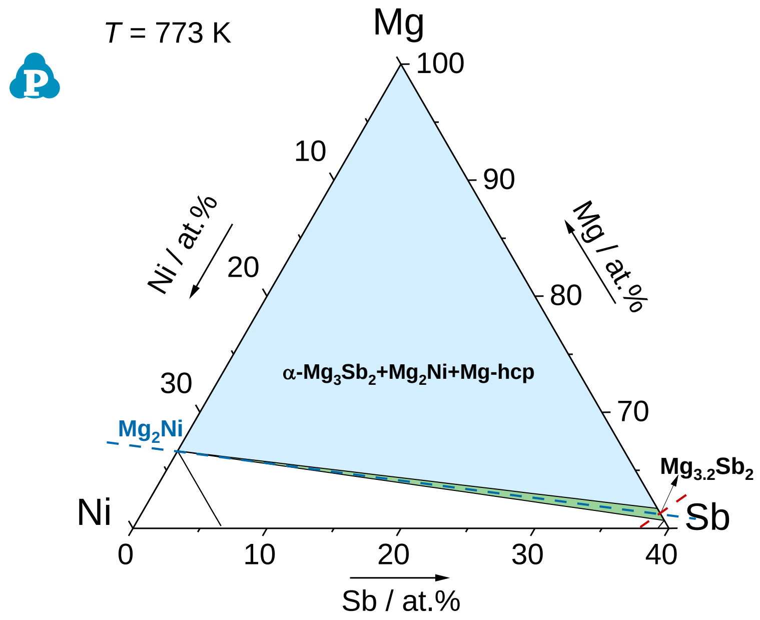

Supplementary Figure 5| The three-phase region of Mg-Mg_2Ni-Mg_3Sb_2 at 773 K.

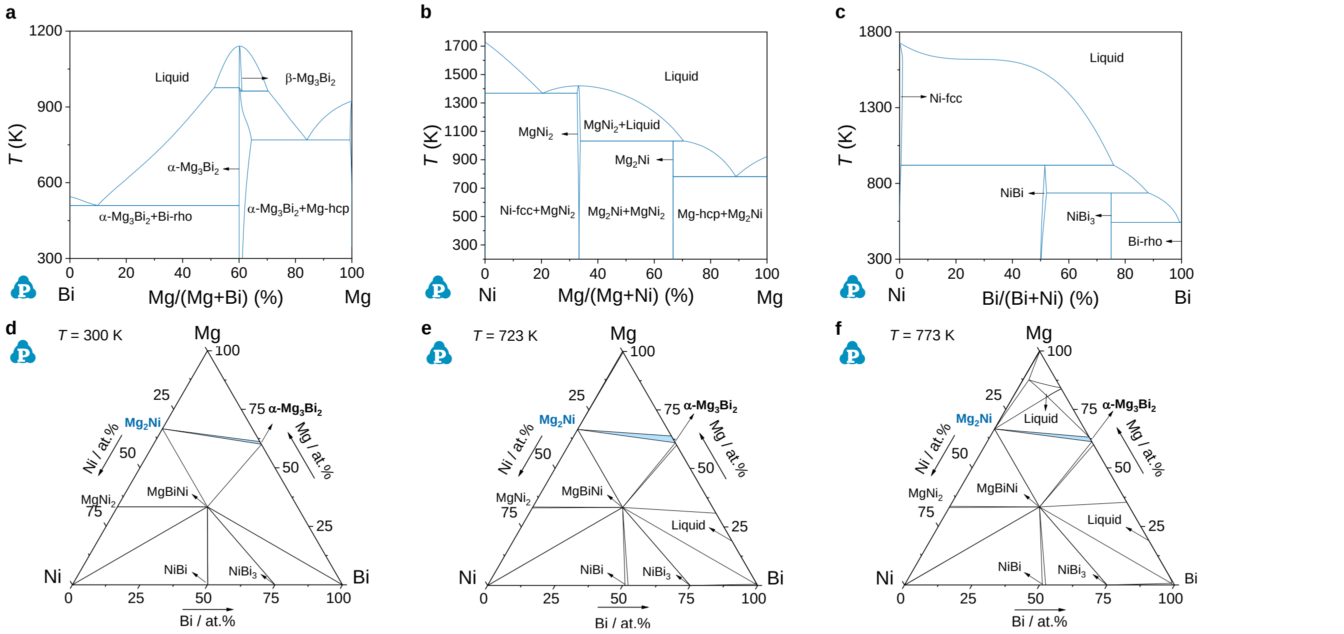

Supplementary Figure 6| The calculated phase diagram. Calculated (a) Mg-Bi binary phase diagram, (b) Mg-Ni binary phase diagram, (c) Ni-Bi binary phase diagram, isothermal sections at (d) 300 K, (c)723 K, and (f) 773 K.

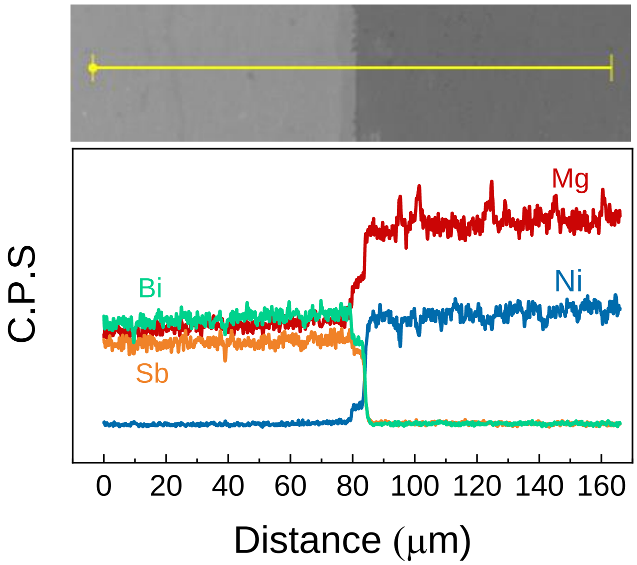

Supplementary Figure 7| SEM images and EDS line scanning results for Mg_2Ni/ $Mg_{3.15}Co_{0.05}SbBi_{0.99}Se_{0.01}$ junction.

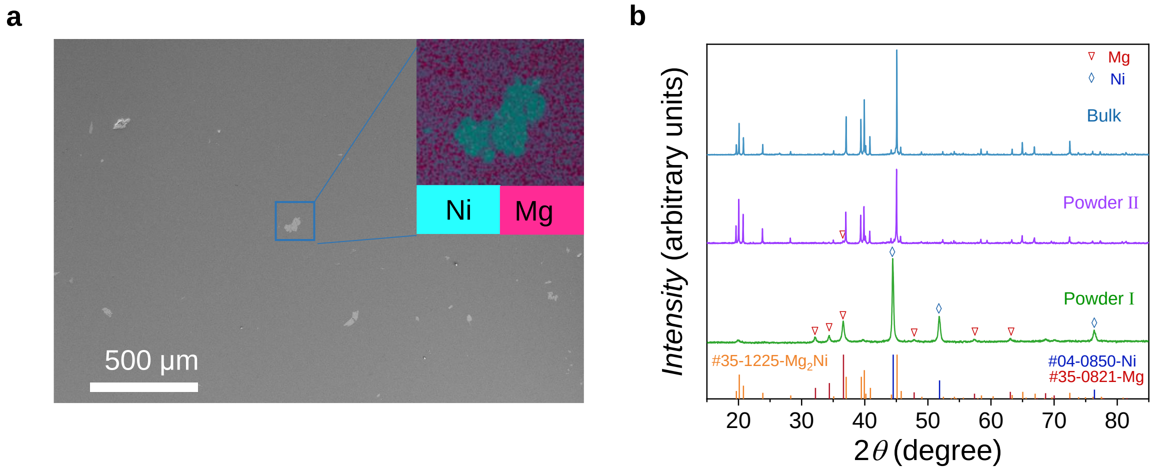

Supplementary Figure 8| Characterizations of Mg_2Ni contact materials. (a) SEM image of sintered Mg_2Ni bulk and (b) XRD patterns of bulk and powders.

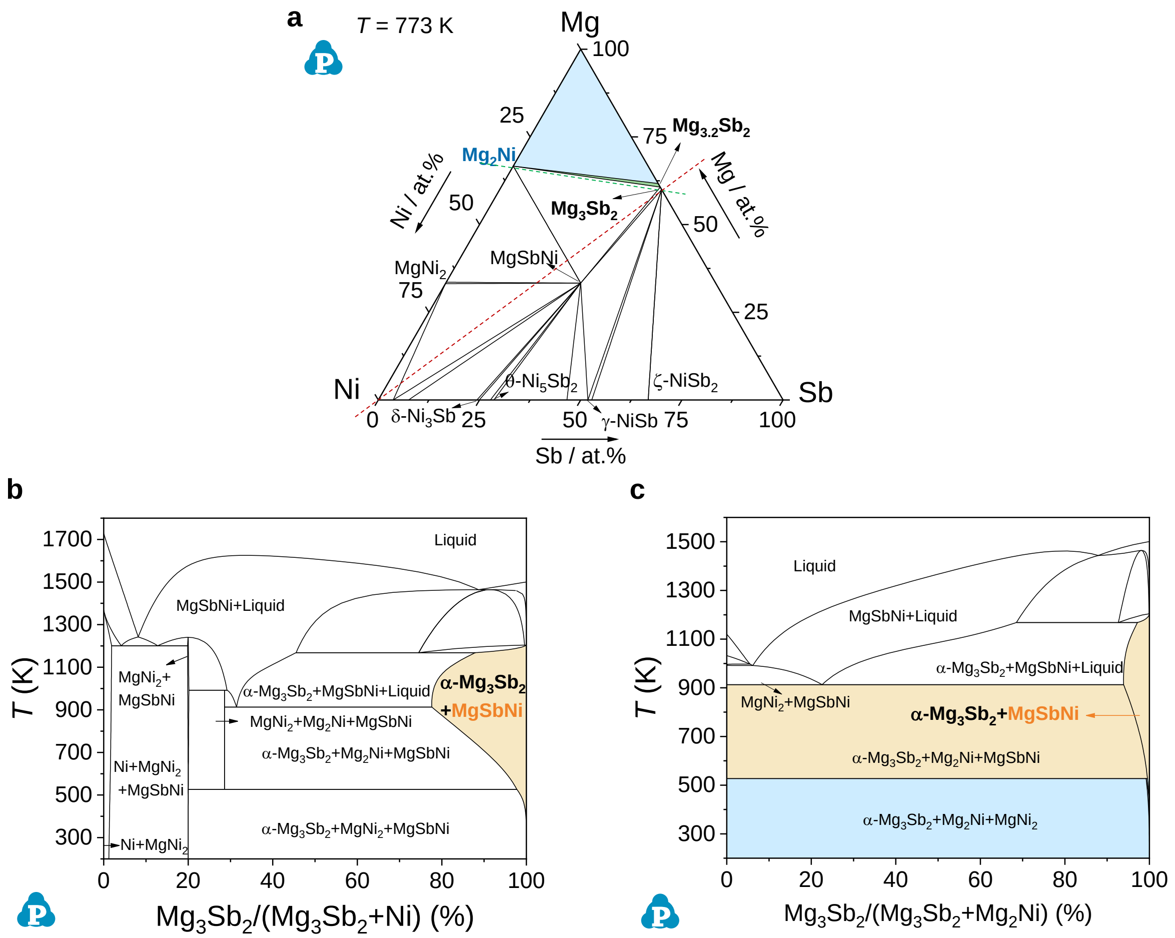

Supplementary Figure 9| The calculated phase diagram. (a) The isothermal section at 773 K of the Ni–Mg–Sb ternary phase diagram. Calculated vertical section of (b) Ni–Mg_3Sb_2 and (c) Mg_2Ni–Mg_3Sb_2.

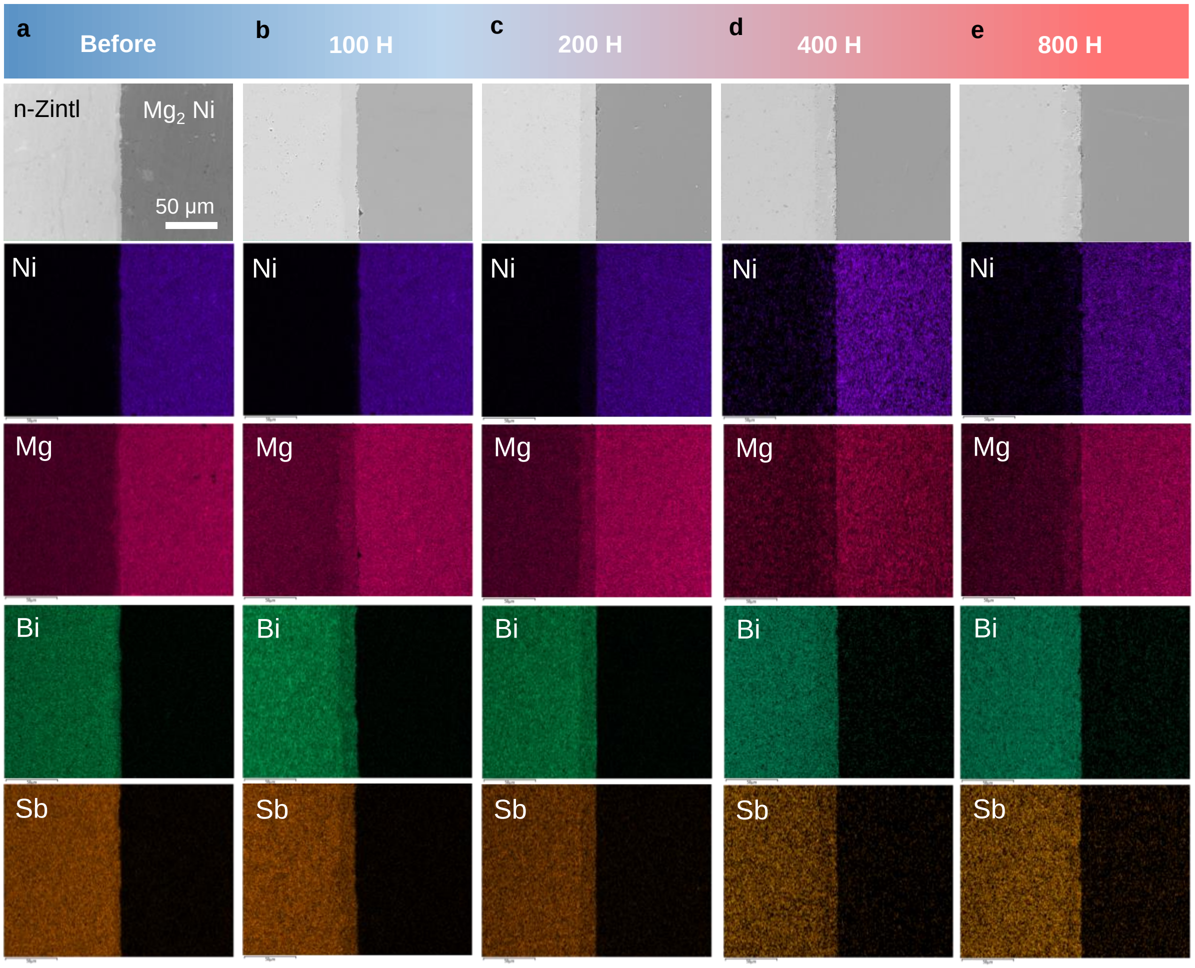

Supplementary Figure 10| Characterizations of $Mg_2Ni/Mg_{3.15}Co_{0.05}SbBi_{0.99}Se_{0.01}$ junctions. SEM images and EDS mapping results of $Mg_2Ni/Mg_{3.15}Co_{0.05}SbBi_{0.99}Se_{0.01}$ junctions (a) as-prepared, and aging at 673 K for (b) 100 hours, (c) 200 hours, (d) 400 hours, and (e) 800 hours.

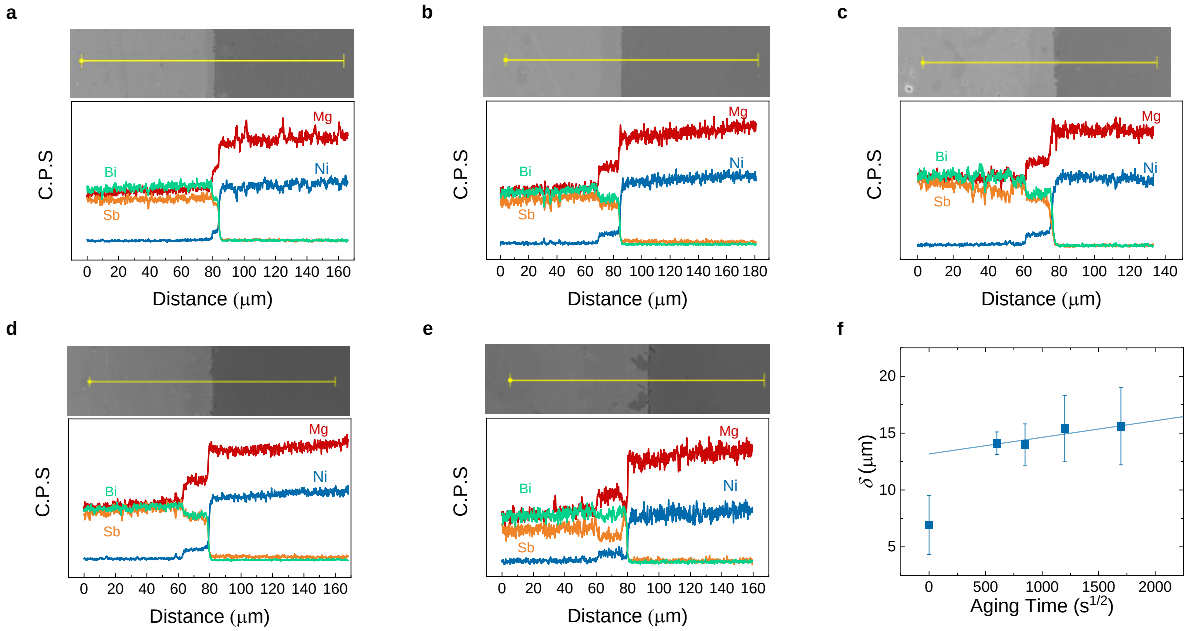

Supplementary Figure 11| Characterizations of $Mg_2Ni/Mg_{3.15}Co_{0.05}SbBi_{0.99}Se_{0.01}$ junctions reaction rate. SEM images and EDS line scanning results of $Mg_2Ni/Mg_{3.15}Co_{0.05}SbBi_{0.99}Se_{0.01}$ junctions (a) as-prepared, and aging at 673 K for (b) 100 hours, (c) 200 hours, (d) 400 hours, and (e) 800 hours. (f) The fitting curves of the thickness of reaction layer of Mg_2Ni junctions aging at 673 K.

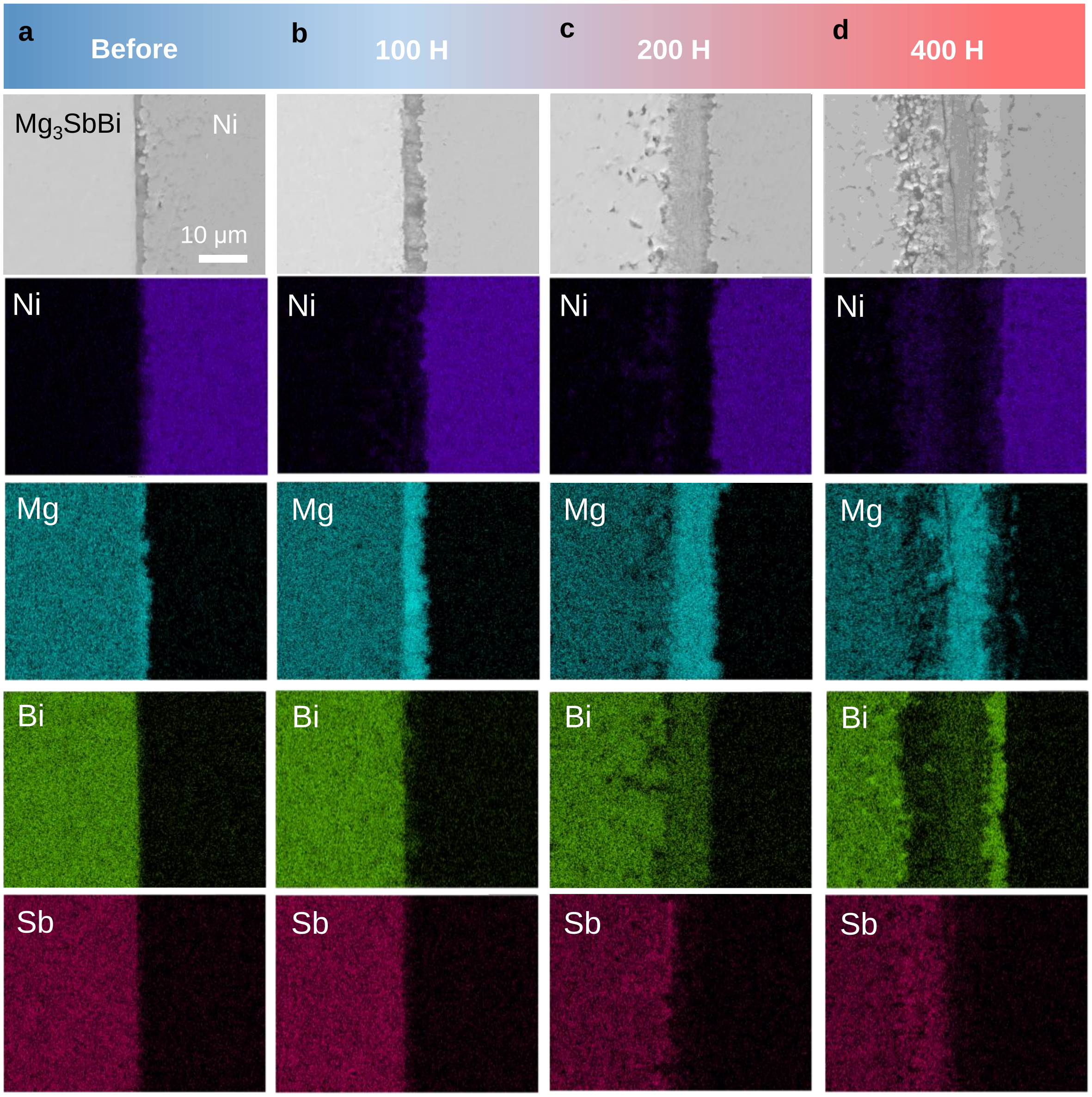

Supplementary Figure 12| Characterizations of Ni/$Mg_{3.15}Co_{0.05}SbBi_{0.99}Se_{0.01}$ junctions. SEM images and EDS mapping results of Ni/$Mg_{3.15}Co_{0.05}SbBi_{0.99}Se_{0.01}$ junctions (a) as-prepared, and aging at 673 K for (b) 100 hours, (c) 200 hours, amd (d) 400 hours.

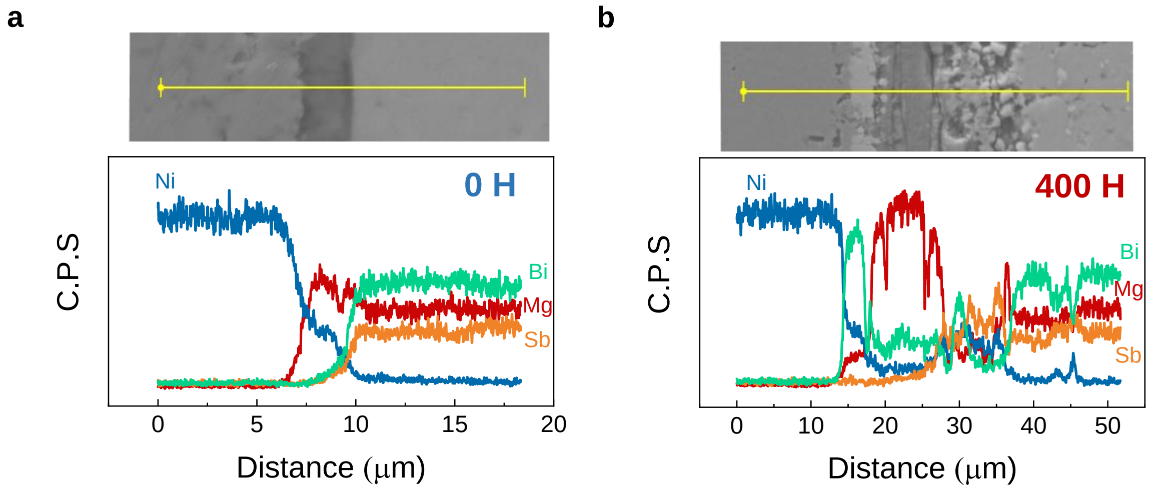

Supplementary Figure 13| Characterizations of Ni/$Mg_{3.15}Co_{0.05}SbBi_{0.99}Se_{0.01}$ junctions reaction rate. SEM images and EDS line scanning results of Ni/$Mg_{3.15}Co_{0.05}SbBi_{0.99}Se_{0.01}$ junctions (a) as-prepared, and aging at 673 K for (b) 400 hours.

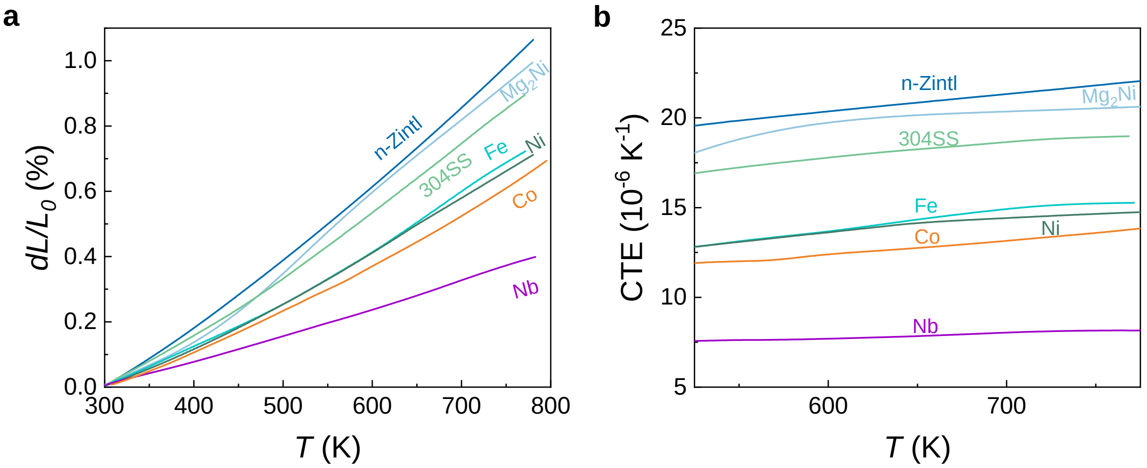

Supplementary Figure 14| Characterization of thermal expansion behavior of metals and alloys. Temperature-dependent expansion behaviors of $Mg_{3.15}Co_{0.05}SbBi_{0.99}Se_{0.01}$, Mg_2Ni, 304 stainless steel, Fe, Ni, Co, and Nb in the range of 293-773 K: (a) dL/L_0 and (b) CTE.

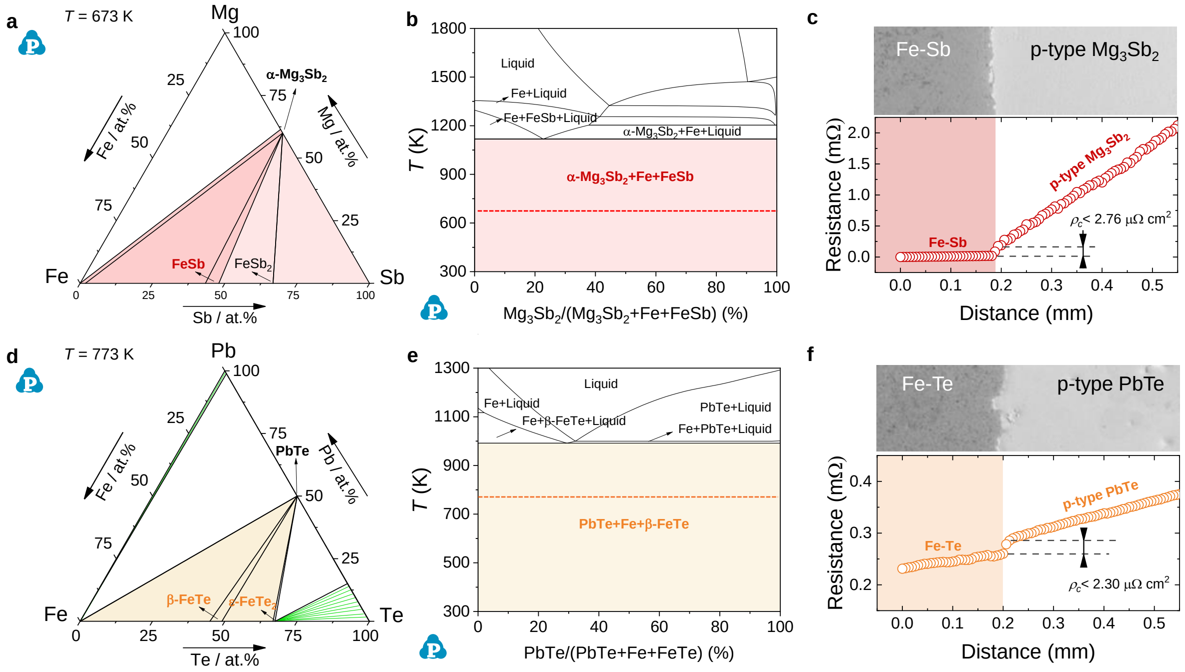

Supplementary Figure 15| The calculated phase diagram in this work and the experimental results. The isothermal sections of (a) Mg-Sb-Fe ternary phase diagram at 673 K and (d) Pb-Te-Fe ternary phase diagram at 773 K. Calculated vertical sections of (b) 70Fe30Sb-Mg_3Sb_2 and (e) 70Fe30Te-PbTe. Surface morphology and measured contact resistivity (ρ_c) of (c) 70Fe30Sb/Mg_3Sb_2 and (f) 70Fe30Te/PbTe junctions.

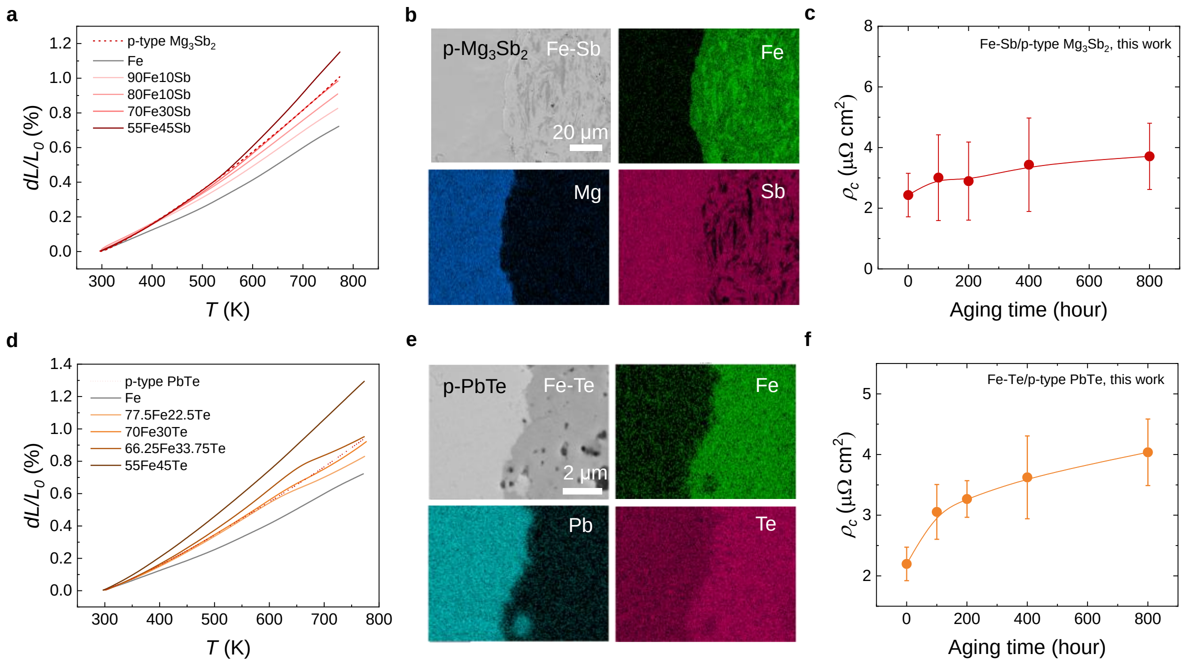

Supplementary Figure 16| Design and characterization of the thermoelectric contact structures. Thermal expansion behavior of (a) Fe-Sb alloys and p-type Mg_3Sb_2 material, and (d) Fe-Te alloys and p-type PbTe material. SEM images and EDS mapping results of (b) 70Fe30Sb/Mg_3Sb_2 junctions and (e) 70Fe30Te/PbTe junctions after 800 hours aging. Corresponding changes in ρ_c of (c) 70Fe30Sb/Mg_3Sb_2 junctions and (f) 70Fe30Te/PbTe junctions with different aging time.

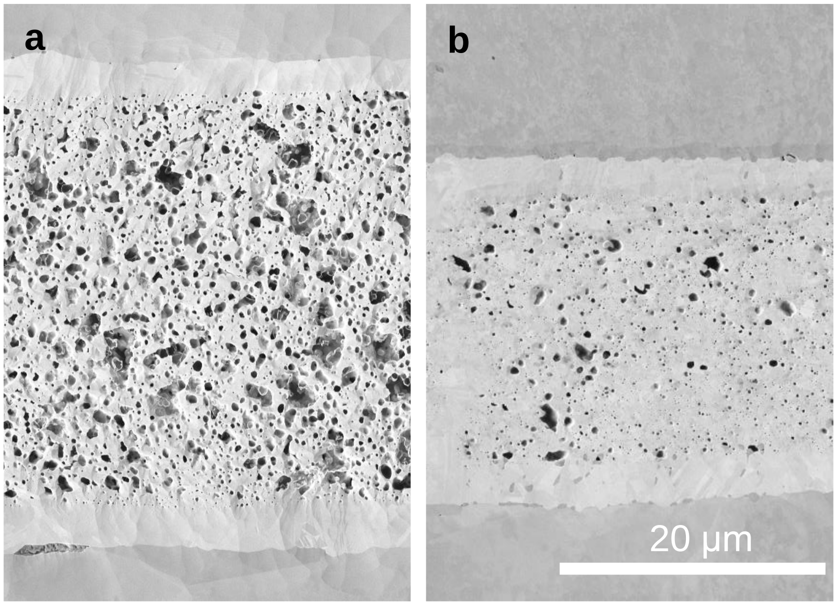

Supplementary Figure 17| Characterization of the nano-silver sintered connection layer. SEM cross-sectional morphologies of Ag NP joints aging after 100 hours at (a) 0.25 MPa and (b) 0.5 MPa.

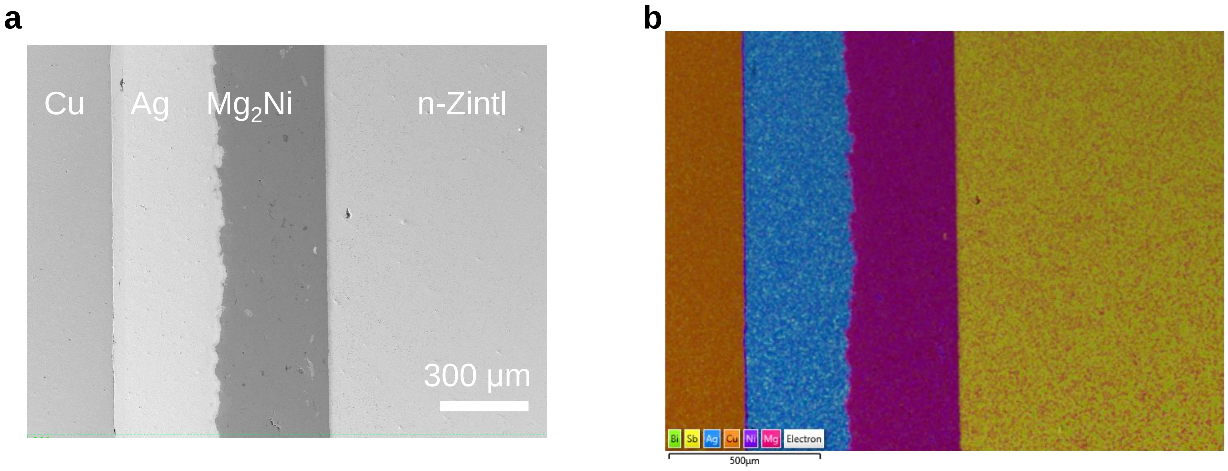

Supplementary Figure 18| Characterizations of n-Zintl junctions. (a) SEM images and (b) EDS mapping results for n-Zintl junctions after Ag NP sintering.

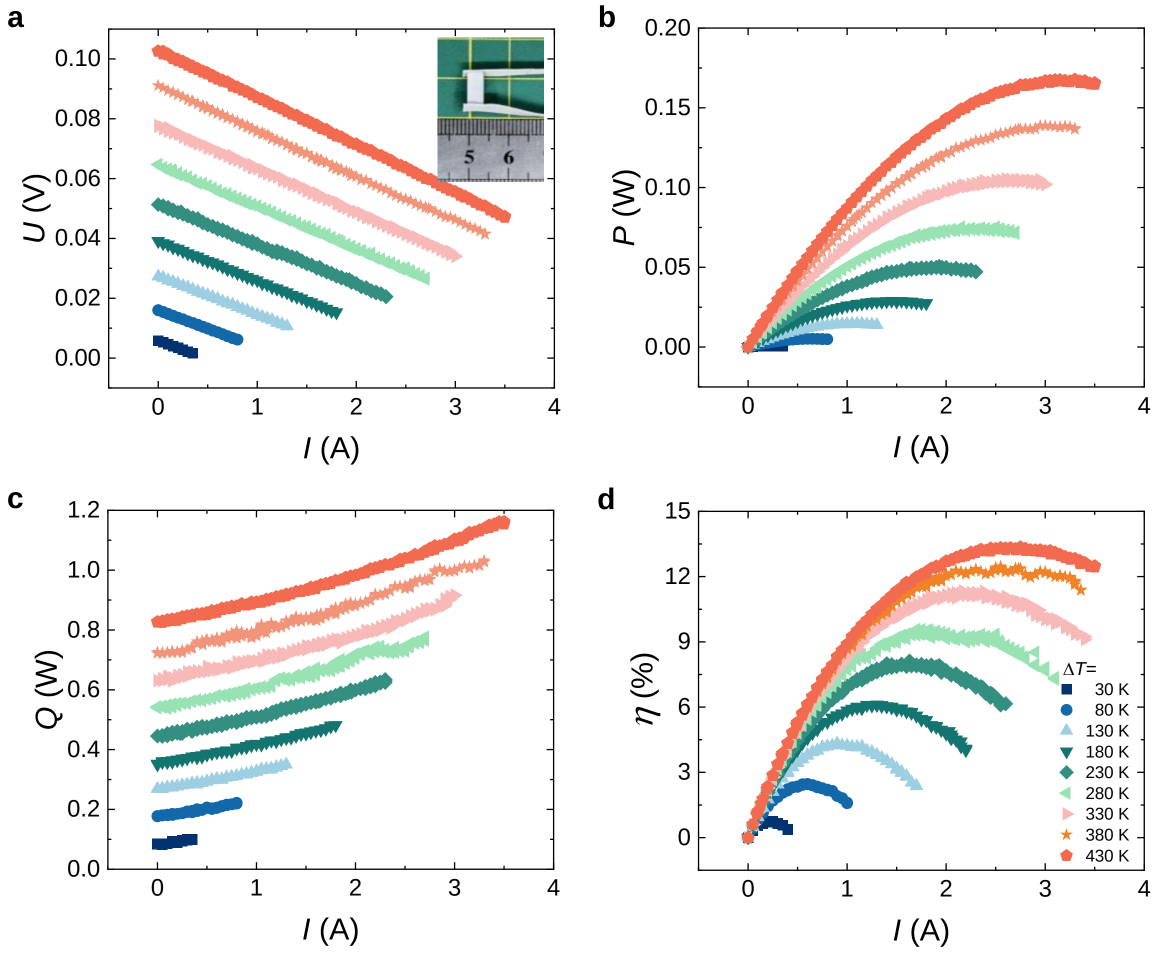

Supplementary Figure 19| Measured properties of n-Zintl single-leg module. Current-dependent (a) output voltage, (b) output power, (c) heat flow, and (d) efficiency for the single-leg device under different temperature gradients.

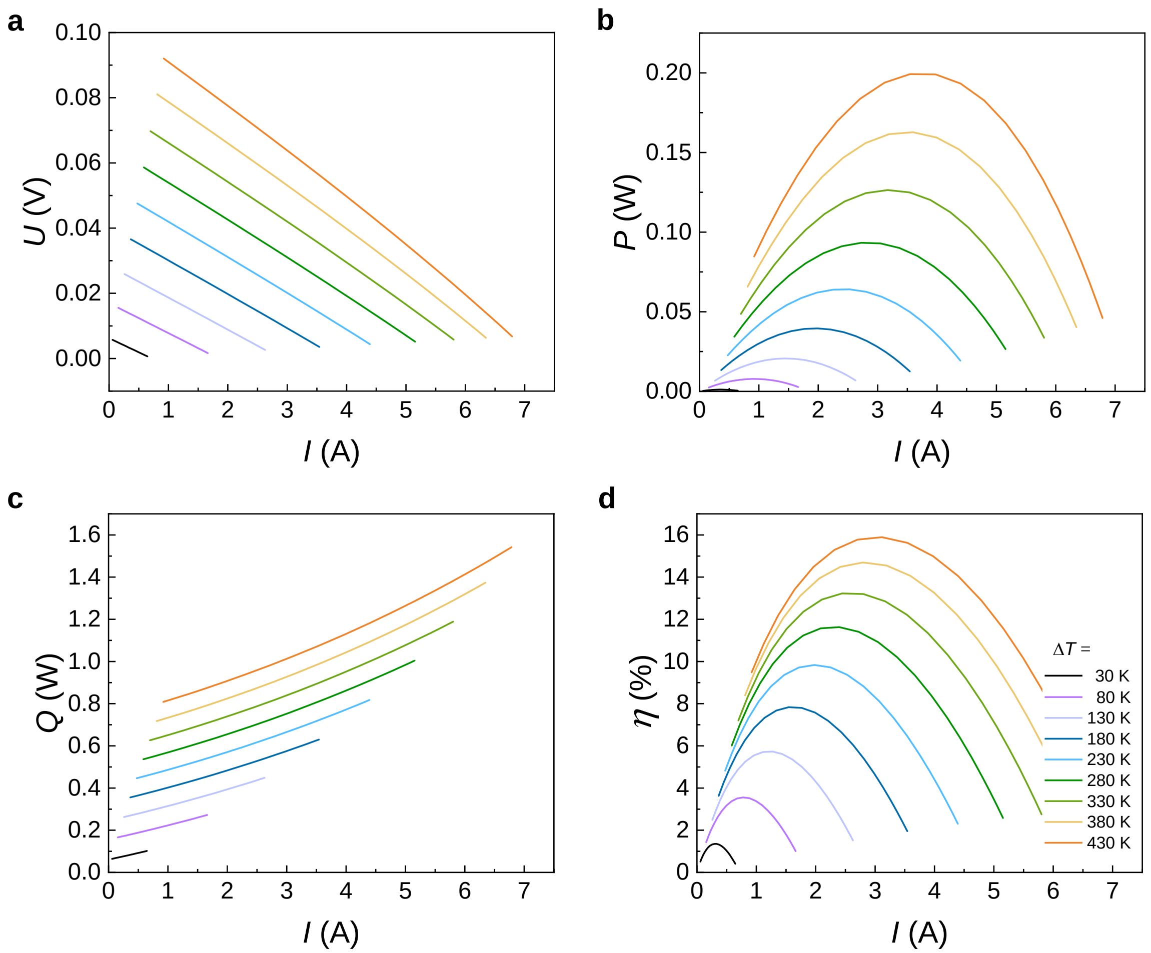

Supplementary Figure 20| Simulated properties of the n-Zintl single-leg module. Current-dependent (a) output voltage, (b) output power, (c) heat flow, and (d) efficiency for the single-leg device under different temperature gradients.

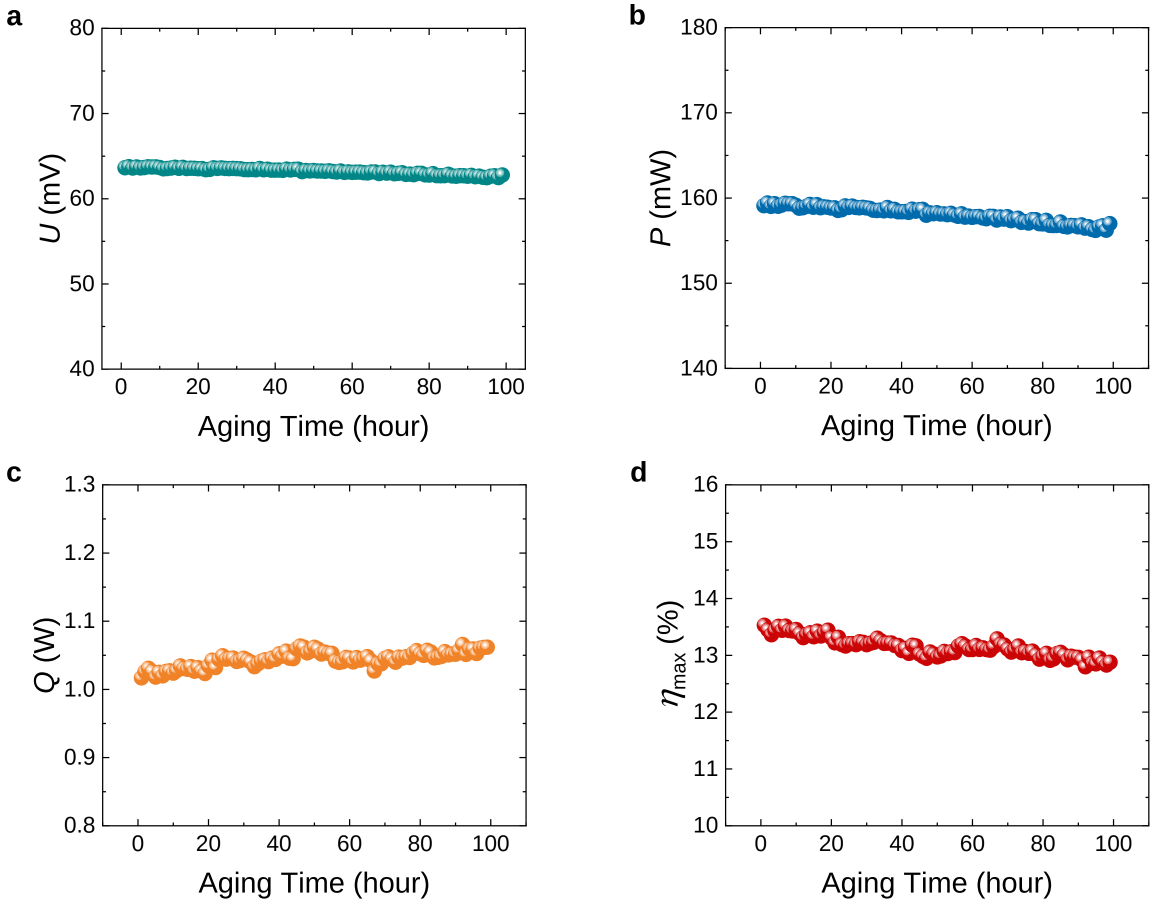

Supplementary Figure 21| Thermal aging test results for n-Zintl single-leg module. Showing changes in (a) output voltage, (b)output power, (c) heat flow, and (d) conversion efficiency as a function of the aging time.

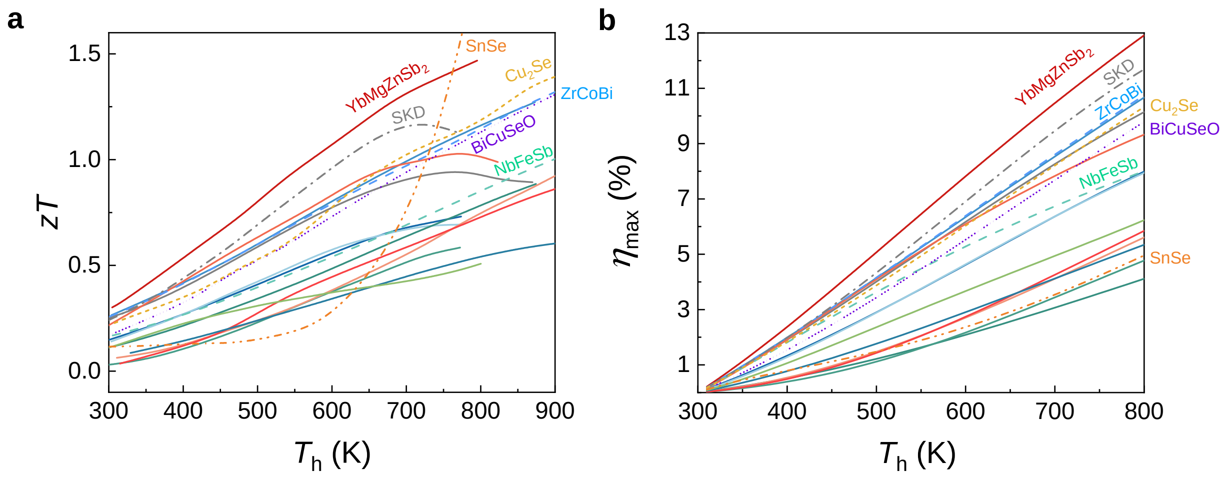

Supplementary Figure 22| Comparison of thermoelectric performance [69-85] and simulated conversion efficiency of p-type TE materials. (a) *zT* and (b) finite-element-simulated maximum efficiency of 18 different p-type TE candidates.

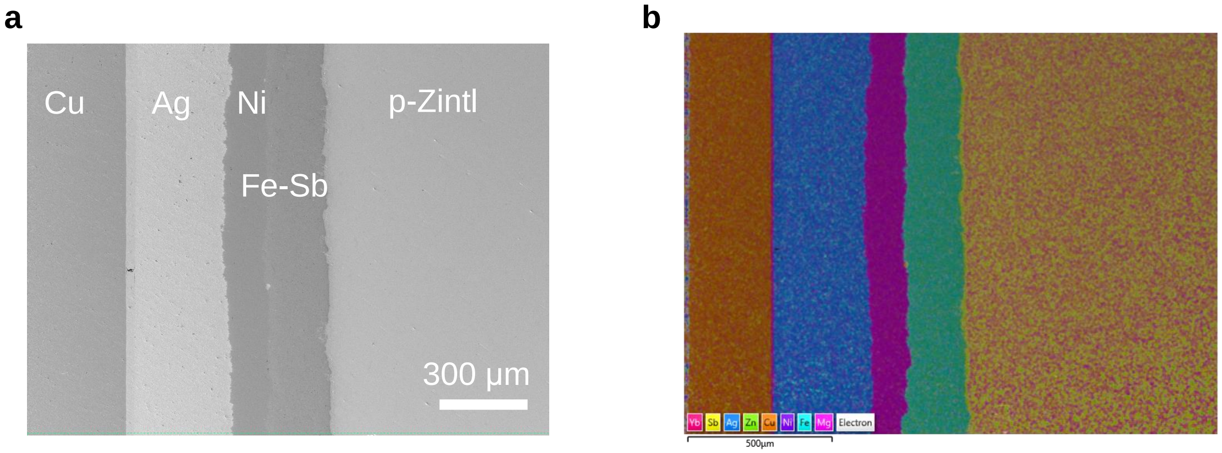

Supplementary Figure 23| Characterizations of p-Zintl junctions. (a) SEM images and (b) EDS mapping results for p-Zintl junctions after Ag NP sintering.

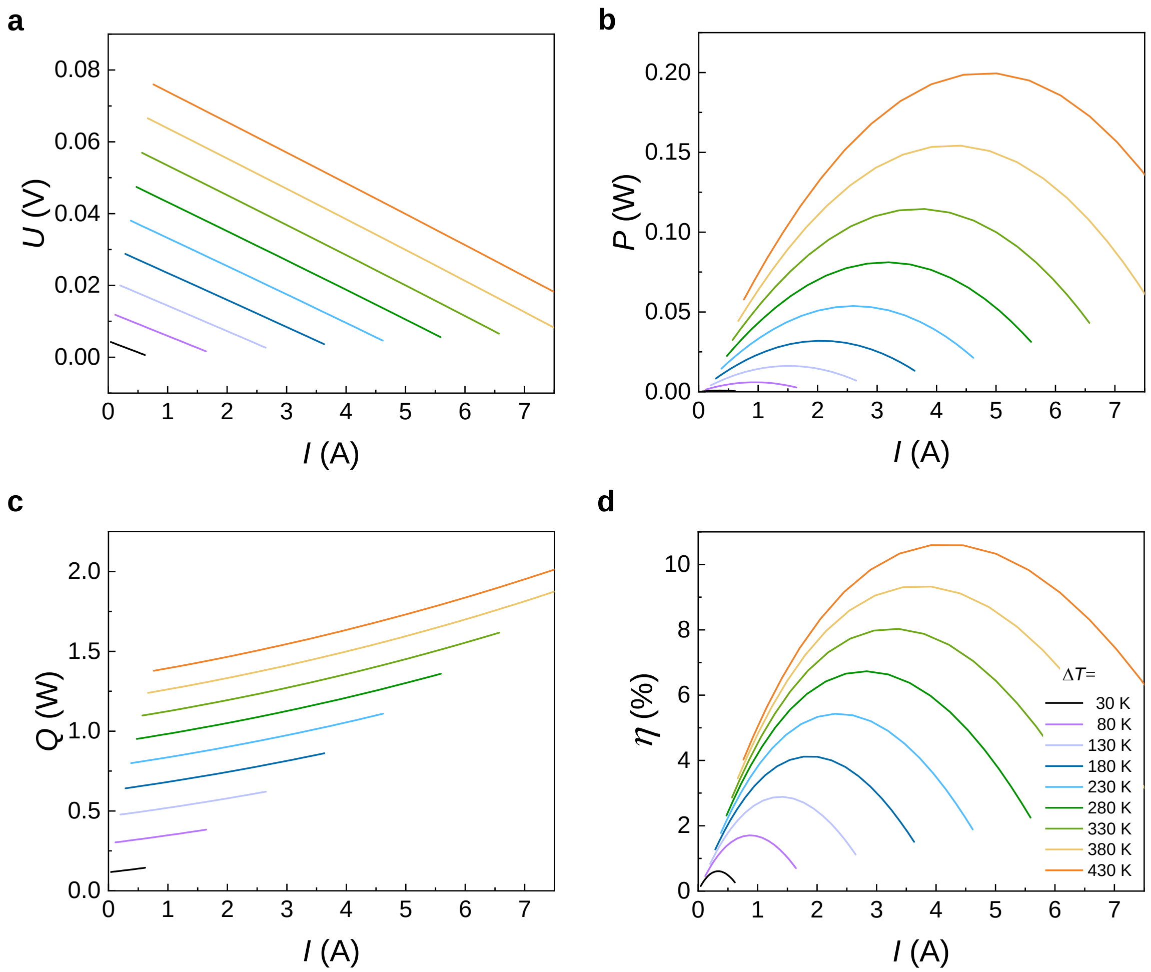

Supplementary Figure 24| Simulated properties of the p-Zintl single-leg module. Current-dependent (a) output voltage, (b) output power, (c) heat flow, and (d) efficiency for the single-leg device under different temperature gradients.

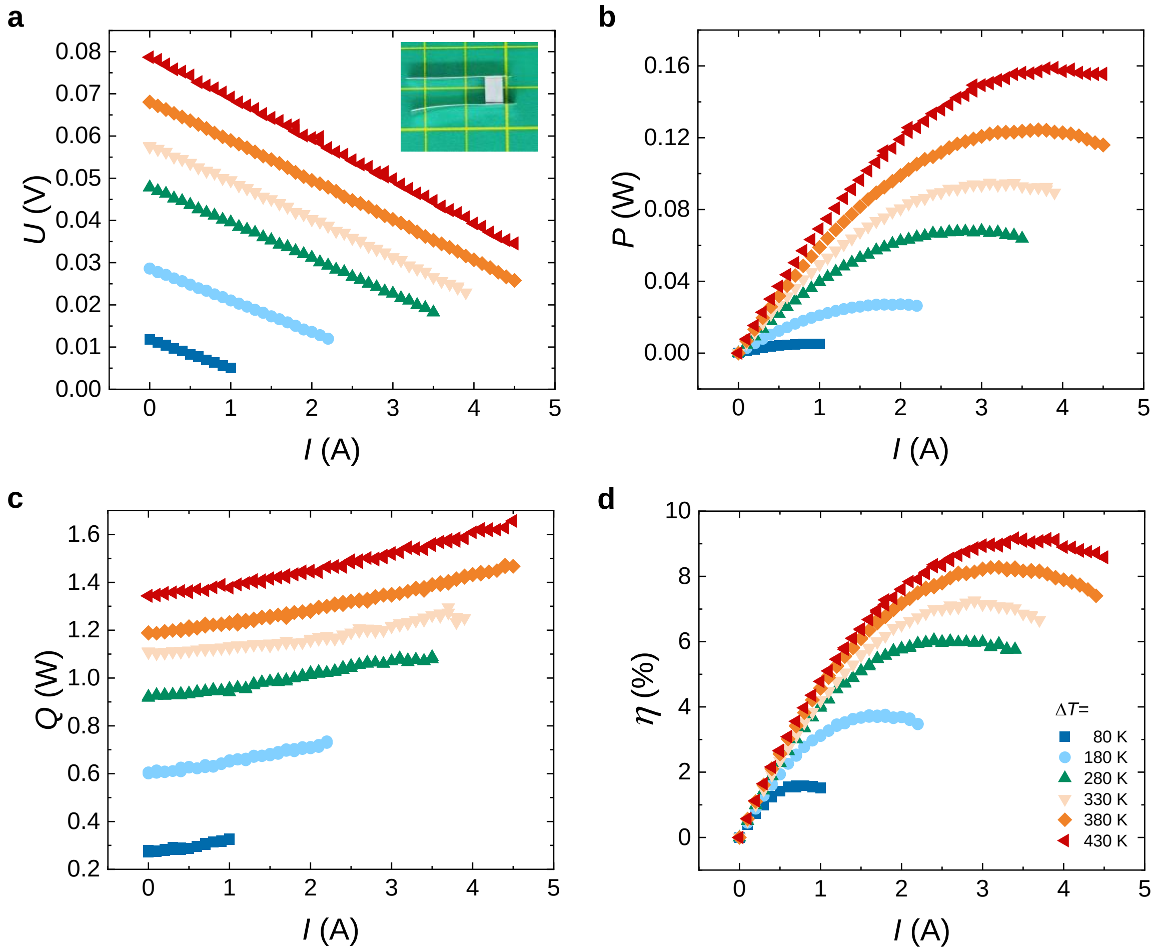

Supplementary Figure 25| Measured properties of p-Zintl single-leg module. Current-dependent (a) output voltage, (b) output power, (c) heat flow, and (d) efficiency for the single-leg device under different temperature gradients.

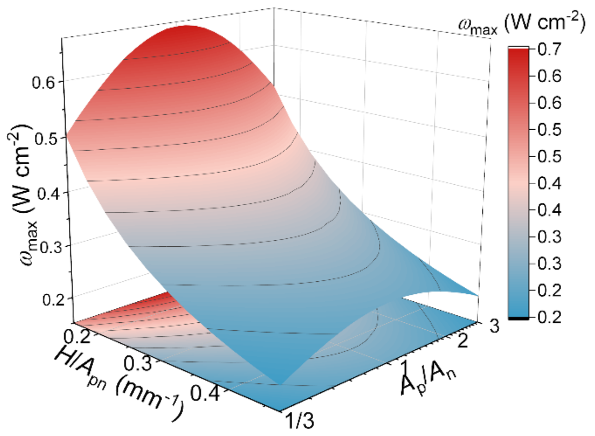

Supplementary Figure 26| Simulated maximum output power density (ω_{max}) as a function of A_p/A_n and H/A_{pn}.

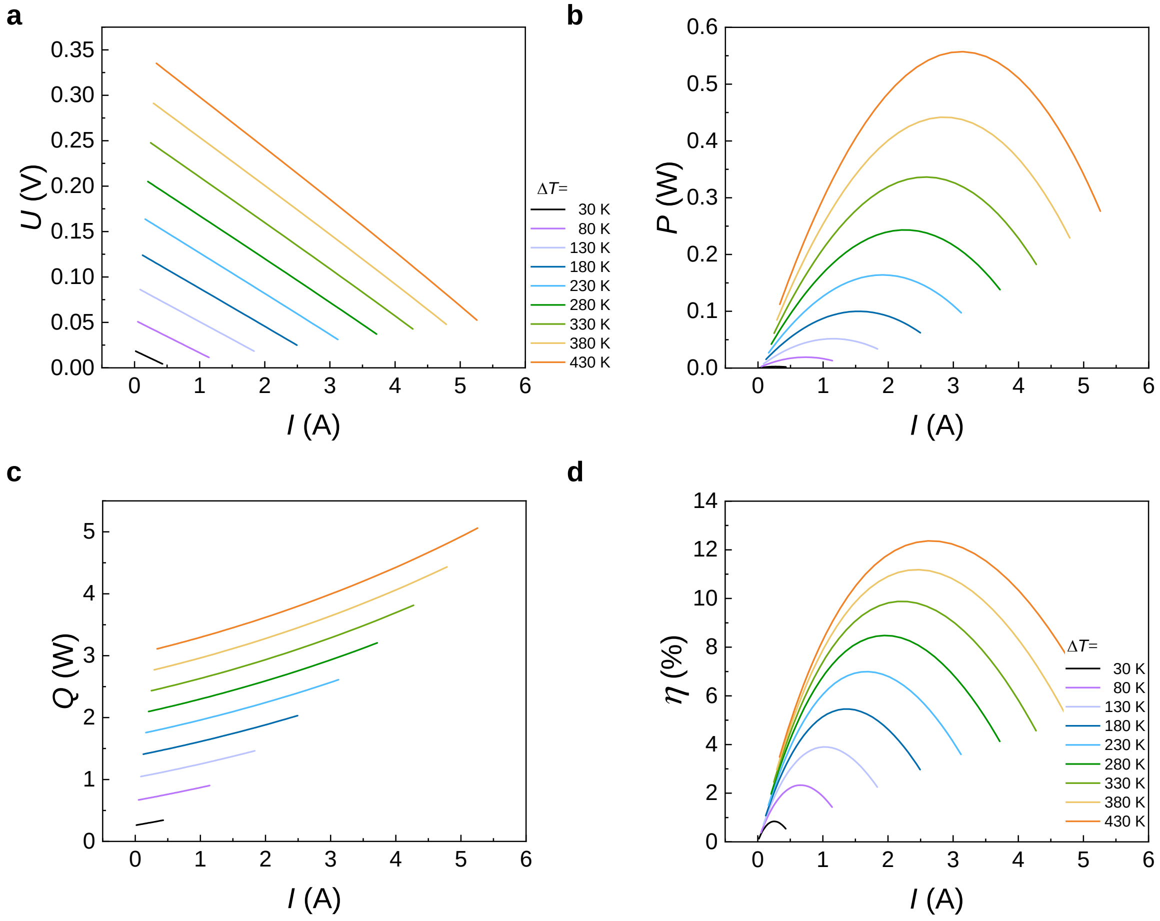

Supplementary Figure 27| Simulated properties of the Zintl-based module ($Mg_{3.15}Co_{0.05}SbBi_{0.99}Se_{0.01}/Yb_{0.9}Mg_{0.9}Zn_{1.198}Ag_{0.002}Sb_2$). Current-dependent (a) output voltage, (b) output power, (c) heat flow, and (d) efficiency for the 2-pair module under different temperature gradients.

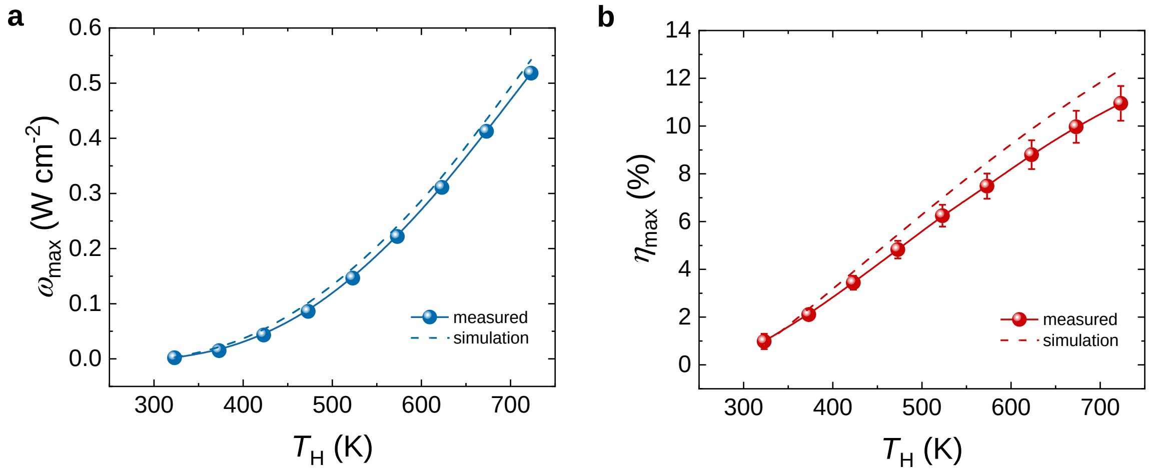

Supplementary Figure 28| Comparison between measured parameters and simulated values of the Zintl-based module ($Mg_{3.15}Co_{0.05}SbBi_{0.99}Se_{0.01}$/$Yb_{0.9}Mg_{0.9}Zn_{1.198}Ag_{0.002}Sb_2$). (a) The maximum power density (ω_{max}) and (b) maximum efficiency (η_{max}) under different temperature gradients.

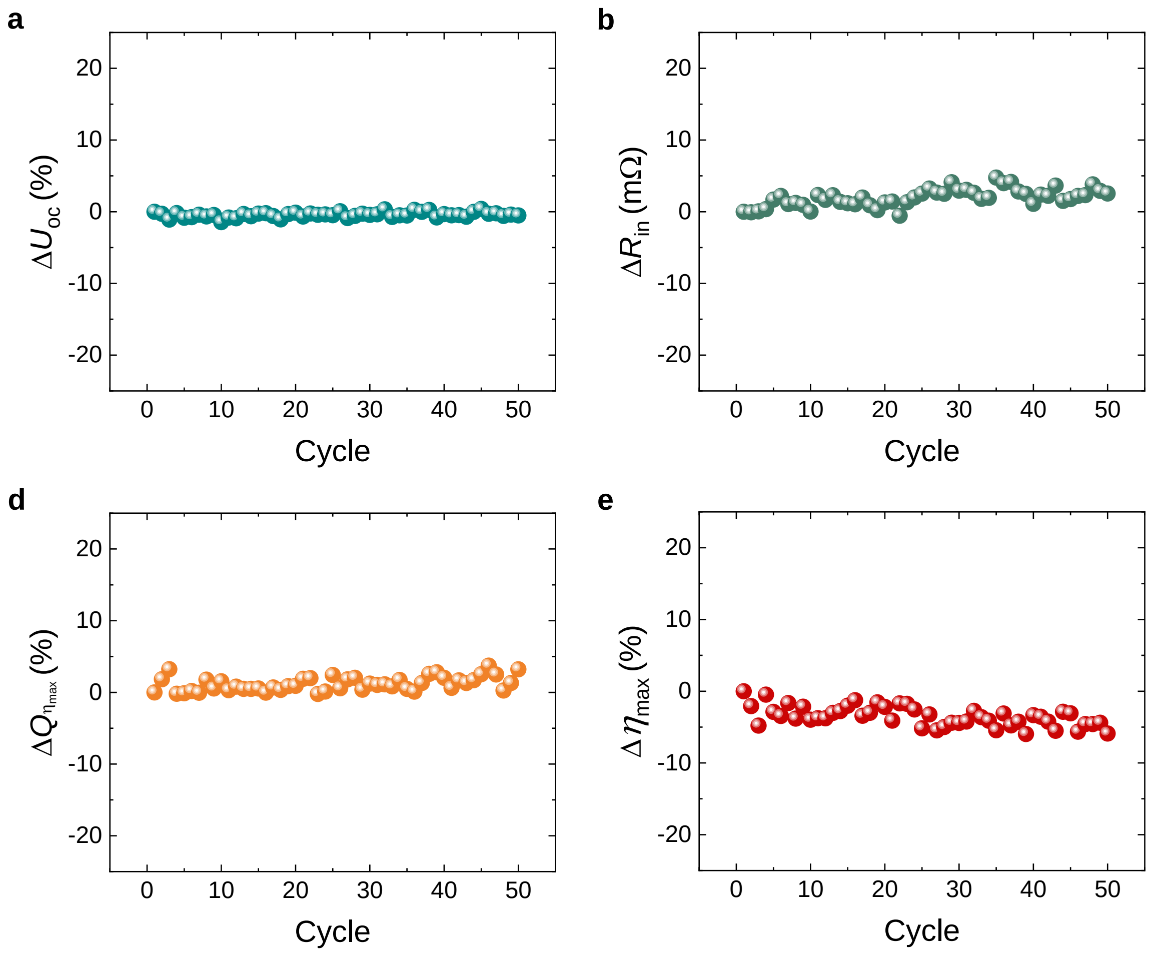

Supplementary Figure 29| Thermal cycling test results for the Zintl-based module ($Mg_{3.15}Co_{0.05}SbBi_{0.99}Se_{0.01}/Yb_{0.9}Mg_{0.9}Zn_{1.198}Ag_{0.002}Sb_2$). showing changes in (a) open circuit voltage, (b) internal resistance, (c) heat flow at maximum efficiency, and (d) maximum efficiency as a function of thermal cycle number.

Supplementary Table 1| Chemical compositions of interlayer determined by EDS.

Element composition	Mg (at.%)	Sb (at.%)	Bi (at.%)	Ni (at.%)
1	64.16	12.48	13.37	9.99
2	64.76	13.13	13.53	8.58
3	64.19	12.16	13.83	9.83
4	64.33	12.48	13.17	10.02
5	65.59	13.02	12.59	8.80
6	64.79	13.62	12.94	8.66
AVG	64.64	13.62	12.94	9.31

Supplementary Table 2| Finite element simulation setting parameters.

Parameter	Value
Total cross-sectional area A_{pn}	4×8 mm^2
Cold-side thermal contact resistance	1.2×10^4 $Wm^{-2}K^{-1}$
Hot-side thermal contact resistance	2×10^3 $Wm^{-2}K^{-1}$
Electrical contact resistivity	10 μΩ·cm^2
Cold-side temperature	293 K
Hot-side temperature	723 K

Supplementary Table 3| Crystallographic data and models for all the involved phases in the Mg-Ni-Sb system.

Phase	Pearson	Space group	Prototype	Model
Liquid	-	-	-	(Mg, Ni, Sb, Mg_3Sb_2)
Mg	*hP*2	$P6_3/mmc$	Mg	$(Mg, Ni, Sb)_1(Va)_1$
Ni	*cF*4	$Fm\bar{3}m$	Cu	$(Mg, Ni, Sb)_1(Va)_{0.5}$
Sb	*hR*2	$R\bar{3}m$	αAs	$(Sb)_1$
α-Mg_3Sb_2	*hP*5	$P\bar{3}$m1	La_2O_3	$(Mg)_3(Sb, Va)_2$
β-Mg_3Sb_2	*cI*80	$Ia\bar{3}$	Mn_2O_3	$(Mg)_6(Sb, Va)_3(Sb)_1$
$MgNi_2$	*hP*24	$P6_3/mmc$	$MgNi_2$	$(Mg, Ni)_1(Ni, Mg)_2$
Mg_2Ni	*hP*18	$P6_222$	Mg_2Ni	$(Mg)_2(Ni)_1$
β-Ni_3Sb	*cF*16	*Pmmm*	Cu_3Ti	$(Sb)_1(Ni, Va)_2(Va, Ni)_1$
δ-Ni_3Sb	*oP*8	*Pmmm*	Cu_3Ti	$(Ni)_3(Ni, Sb)_1$
θ-Ni_5Sb_2	-	-	Fe_3Si	$(Ni)_5(Ni, Sb)_2$
γ-NiSb	*hP*4	$P6_3/mmc$	NiAs	$(Sb)_1(Ni, Va)_1(Va, Ni)_1$
ζ-$NiSb_2$	*oP*6	*Pnmm*	FeS_2	$(Ni)_1(Sb)_2$
MgNiSb	*cF12*	$F\bar{4}3m$	MgAgAs	$(Mg)_1(Ni)_1(Sb)_1$

(Va means vacancy)

Supplementary Table 4| Parameters for calculation of phase equilibria in Mg-Ni-Sb system.

**Thermodynamic parameters**	**Ref.**
Liquid: (Mg, Ni, Sb, Mg_3Sb_2)$_1$	
(0)L(Liquid, Mg, Sb) = -64574.5-13.8*T	[48]
(1)L(Liquid, Mg, Sb) = -1*T	[48]
(0)L(Liquid, Mg_3Sb_2) = -261367+125.8*T-9.85*T*ln(T)+3*G_{Mg}^{Liq}+2*G_{Sb}^{Liq}	[48]
(0)L(Liquid, Mg, Mg_3Sb_2) = -14941.2-14.3663*T	[48]
(1)L(Liquid, Mg, Mg_3Sb_2) = +10399.95+3*T	[48]
(0)L(Liquid, Mg_3Sb_2, Sb) = -19774.6-21.997*T	[48]
(1)L(Liquid, Mg_3Sb_2, Sb) = -17802.3+3.3426*T	[48]
(0)L(Liquid, Ni, Sb) = -73580.86-4.01441*T	[49]
(1)L(Liquid, Ni, Sb) = -12843.3-11.0286*T	[49]
(2)L(Liquid, Ni, Sb) = -4010+9.7*T	[49]
(0)L(Liquid, Mg, Ni) = -50910.00+25.79995*T	[45]
(1)L(Liquid, Mg, Ni) = -14989.95+13.24788*T	[45]
(0)L(Liquid, Mg, Ni, Sb) = -146000	[*]
Fcc_A1: (Mg, Ni, Sb)$_1$(Va)$_1$	
(0)L(Fcc_A1, Mg, Ni: Va) = +100000	[47]
(0)L(Fcc_A1, Ni, Sb: Va) = -64860+17.119*T	[49]
(1)L(Fcc_A1, Ni, Sb: Va) = -33663.84+4.5*T	[49]
(2)L(Fcc_A1, Ni, Sb: Va) = -38039.58+3.034*T	[49]
(0)L(Fcc_A1, Mg, Ni, Sb: Va) = +500000	[*]
Hcp_A3: (Mg, Ni, Sb)$_1$(Va)$_{0.5}$	
(0)L(Hcp_A3, Mg, Ni: Va) = +100000	[45]
(0)L(Hcp_A3, Mg, Sb: Va) = -61998.6-18.999*T	[48]
(0)L(Hcp_A3, Ni, Sb: Va) = +5000	[49]
(0)L(Hcp_A3, Mg, Ni, Sb: Va) = +500000	[*]
Alpha-Mg_3Sb_2: (Mg)$_3$(Sb, Va)$_2$	
(0)L(Alpha-Mg_3Sb_2, Mg: Sb) = -294509-14.05*T + 11.212*T*ln(T)-0.013*T**2 + 3*G_{Mg}^{Ser}+2*G_{Sb}^{Ser}	[48]
(0)L(Alpha-Mg_3Sb_2, Mg: Va) = +40665.1+9*T+3*G_{Mg}^{Ser}	[48]
(0)L(Alpha-Mg_3Sb_2, Mg: Sb, Va) = -13675.1+5.432*T	[48]
(1)L(Alpha-Mg_3Sb_2, Mg: Sb, Va) = -14000+7.476*T	[48]
Beta-Mg_3Sb_2: (Mg)$_6$(Sb, Va)$_3$(Sb)$_1$	
(0)L(Beta-Mg_3Sb_2, Mg: Sb: Sb) = -386039-190.64*T+17.16*T*ln(T)+6*G_{Mg}^{Ser}+4*G_{Sb}^{Ser}	[48]
(0)L(Beta-Mg_3Sb_2, Mg: Va: Sb) = -33000.4-15.1*T+14.25*T*ln(T)+6*G_{Mg}^{Ser}+1*G_{Sb}^{Ser}	[48]
(0)L(Beta-Mg_3Sb_2, Mg: Sb, Va: Sb) = -68212.8-29.878*T	[48]

(1)L(Beta-Mg_3Sb_2, Mg: Sb, Va: Sb) = -106813-28.0*T	[48]
Mg_2Ni: $(Mg)_{0.6667}(Ni)_{0.3333}$	
(0)L(Mg_2Ni, Mg: Ni) = -20320.4+138.49311*T-24.9354*T*ln(T)-1.538E-3*T**2+133805*T**(-1)	[46]
$MgNi_2$: $(Mg, Ni)_{0.3333}(Mg, Ni)_{0.6667}$	
(0)L($MgNi_2$, Mg: Ni) = -24688.46+147.27013*T-25.7998*T*ln(T)-2.46496E-3*T**2+111575.5*T**(-1)	[46]
(0)L($MgNi_2$, Mg: Mg) = +764.7058861 + 20.5882353*T+1*G_{Mg}^{Ser}	[46]
(0)L($MgNi_2$, Ni: Mg) = +1000+0.3333*G_{Ni}^{Ser}+0.6667*G_{Mg}^{Ser}	[46]
(0)L($MgNi_2$, Ni: Ni) = +5254.902+6.862745*T+1*G_{Ni}^{Ser}	[46]
Beta-Ni_3Sb: $(Sb)_{0.25}(Ni, Va)_{0.5}(Va, Ni)_{0.25}$	
(0)L(Beta-Ni_3Sb, Sb: Ni: Ni) = -19150-3.3*T+0.75*G_{Ni}^{Ser}+0.25*G_{Sb}^{Ser}	[49]
(0)L(Beta-Ni_3Sb, Sb: Ni: Va) = -17522.75-1.74087*T+0.5*G_{Ni}^{Ser}+0.25*G_{Sb}^{Ser}	[49]
(0)L(Beta-Ni_3Sb, Sb: Va: Ni) = -6235.57+8.93666358*T+0.25*G_{Ni}^{Ser}+0.25*G_{Sb}^{Ser}	[49]
(0)L(Beta-Ni_3Sb, Sb: Va: Va) = +5312+19.5*T+0.25*G_{Sb}^{Ser}	[49]
(0)L(Beta-Ni_3Sb, Sb: Ni, Va: Va) = +2000-9.88685*T	[49]
(0)L(Beta-Ni_3Sb, Sb: Ni, Va: Ni) = +2000-9.88685*T	[49]
(0)L(Beta-Ni_3Sb, Sb: Ni: Va, Ni) = -7508.25+8.78*T	[49]
(0)L(Beta-Ni_3Sb, Sb: Va: Va, Ni) = -7508.25+8.78*T	[49]
Gamma-NiSb: $(Sb)_{0.3333}(Ni, Va)_{0.3333}(Va, Ni)_{0.3333}$	
(0)L(Gamma-NiSb, Sb: Ni: Va) = -23310+19.82*T-3.50035*T*ln(T)+0.00932581948*T**2-3.009E-6*T**3+7.414E-21*T**7+0.3333*G_{Ni}^{Ser}+0.3333*$G_{Sb}^{Ser\_Low}$	[49]
(0)L(Gamma-NiSb, Sb: Ni: Ni) = -16000+2*T+0.6667*G_{Ni}^{Ser}+0.3333*G_{Sb}^{Ser}	[49]
(0)L(Gamma-NiSb, Sb: Va: Va) = +5522.55+1.3*T+0.3333*G_{Sb}^{Ser}	[49]
(0)L(Gamma-NiSb, Sb: Va: Ni) = -3182.15338+52.79*T+0.3333*G_{Ni}^{Ser}+0.33333*G_{Sb}^{Ser}	[49]
(0)L(Gamma-NiSb, Sb: Ni, Va: Va) = -1200-2*T	[49]
(0)L(Gamma-NiSb, Sb: Ni, Va: Ni) = -1200-2*T	[49]
(0)L(Gamma-NiSb, Sb: Ni: Va, Ni) = 13500+3*T	[49]
(0)L(Gamma-NiSb, Sb: Va: Va, Ni) = 13500+3*T	[49]
Delta-Ni_3Sb: $(Ni)_{0.75}(Ni, Sb)_{0.25}$	
(0)L(Delta-Ni_3Sb, Ni: Ni) = +3500-1.11*T+G_{Ni}^{Ser}	[49]
(0)L(Delta-Ni_3Sb, Ni: Sb) = -21460-1.31*T+0.75*G_{Ni}^{Ser}+0.25*G_{Sb}^{Ser}	[49]
(0)L(Delta-Ni_3Sb, Ni: Ni, Sb) = -7252.56+11*T	[49]
Theta-Ni_5Sb_2: $(Ni)_{0.7143}(Ni, Sb)_{0.2857}$	
(0)L(Theta-Ni_5Sb_2, Ni: Ni) = +2201.1+0.8005*T+G_{Ni}^{Ser}	[49]

(0)L(Theta-Ni_5Sb_2, Ni: Sb) = -23928.52-1.1*T+0.7143*G_{Ni}^{Ser}+0.2857*G_{Sb}^{Ser}	[49]
(0)L(Theta-Ni_5Sb_2, Ni: Ni, Sb) = -7150+6*T	[49]
Zeta-$NiSb_2$: $(Ni)_{0.3333}(Sb)_{0.6667}$	
(0)L(Zeta-$NiSb_2$, Ni: Sb) = -26020+4.43*T+0.3333*G_{Ni}^{Ser}+0.6667*G_{Sb}^{Ser}	[49]
MgNiSb: $(Mg)_1(Ni)_1(Sb)_1$	
(0)L(MgNiSb, Mg: Ni: Sb) = -150000+2.7*T+G_{Mg}^{Ser}+G_{Ni}^{Ser}+G_{Sb}^{Ser}	[*]

[∗]-this work

Supplementary Table 5| Crystallographic data and models for all the involved phases in the Mg-Ni-Bi system.

Phase	Pearson	Space group	Prototype	Model
Liquid	-	-	-	(Mg, Ni, Bi, Mg_3Bi_2)
Mg	*hP*2	$P6_3/mmc$	Mg	$(Mg, Ni, Sb)_1(Va)_1$
Ni	*cF*4	$Fm\bar{3}m$	Cu	$(Mg, Ni, Sb)_1(Va)_{0.5}$
Bi	*hR*2	$R\bar{3}m$	αAs	$(Bi)_1$
α-Mg_3Bi_2	*hP*5	$P\bar{3}$m1	La_2O_3	$(Mg)_3(Bi, Va)_2$
β-Mg_3Bi_2	-	$R\bar{3}c$	Al_2O_3	$(Mg)_6(Bi, Va)_3(Bi)_1$
$MgNi_2$	*hP*24	$P6_3/mmc$	$MgNi_2$	$(Mg, Ni)_1(Ni, Mg)_2$
Mg_2Ni	*hP*18	$P6_222$	Mg_2Ni	$(Mg)_2(Ni)_1$
Bi_3Ni	*oP*16	*Pnma*	$RhBi_3$	$(Bi)_{0.75}(Ni)_{0.25}$
BiNi	*hP*4	$P6_3/mmc$	-	$(Bi)_1(Ni)_1(Bi, Va)_1$
MgNiBi	*cF12*	$F\bar{4}3m$	MgAgAs	$(Mg)_1(Ni)_1(Bi)_1$

(Va means vacancy)

Supplementary Table 6| Parameters for calculation of phase equilibria in Mg-Ni-Bi system.

Thermodynamic parameters	Ref.
Liquid: (Mg, Ni, Bi, Mg_3Bi_2)$_1$	
(0)L(Liquid, Mg, Bi) = -42939.67-13.82*T	[53]
(1)L(Liquid, Mg, Bi) = +12*T	[53]
(0)L(Liquid, Mg_3Bi_2) = -110200+3*T-2.9*T*ln(T)+3*G_{Mg}^{Liq}+2*G_{Bi}^{Liq}	[53]
(0)L(Liquid, Mg, Mg_3Bi_2) = -37000+20*T	[53]
(1)L(Liquid, Mg, Mg_3Bi_2) = -10000	[53]
(0)L(Liquid, Mg_3Bi_2, Bi) = -36000	[53]
(1)L(Liquid, Mg_3Bi_2, Bi) = +10000	[53]
(0)L(Liquid, Ni, Bi) = -6438+13.2882*T	[54]
(1)L(Liquid, Ni, Bi) = -11315-1.4574*T	[54]
(0)L(Liquid, Mg, Ni) = -50910.00+25.79995*T	[45]
(1)L(Liquid, Mg, Ni) = -14989.95+13.24788*T	[45]
(0)L(Liquid, Mg, Ni, Bi) = -123000	[*]
Fcc_A1: (Mg, Ni, Bi)$_1$(Va)$_1$	
(0)L(Fcc_A1, Mg, Ni: Va) = +100000	[47]
(0)L(Fcc_A1, Ni, Bi: Va) = +20000+12.5*T	[54]
(0)L(Fcc_A1, Mg, Ni, Bi: Va) = +300000	[*]
Hcp_A3: (Mg, Ni, Bi)$_1$(Va)$_{0.5}$	
(0)L(Hcp_A3, Mg, Ni: Va) = +100000	[45]
(0)L(Hcp_A3, Mg, Bi: Va) = -36800-18*T	[54]
(0)L(Hcp_A3, Mg, Ni, Bi: Va) = +300000	[*]
Alpha-Mg_3Bi_2: (Mg)$_3$(Bi, Va)$_2$	
(0)L(Alpha-Mg_3Bi_2, Mg: Bi) = -172400+80.83*T-10*T*ln(T) +3*G_{Mg}^{Ser}+2*G_{Bi}^{Ser}	[53]
(0)L(Alpha-Mg_3Bi_2, Mg: Va) = +60000-17*T+3*G_{Mg}^{Ser}	[53]
(0)L(Alpha-Mg_3Bi_2, Mg: Bi, Va) = -38500+7.8*T	[53]
(1)L(Alpha-Mg_3Bi_2, Mg: Bi, Va) = -38772.7+40*T	[53]
(2)L(Alpha-Mg_3Bi_2, Mg: Bi, Va) = +15500+20*T	[53]
Beta-Mg_3Bi_2: (Mg)$_6$(Bi, Va)$_3$(Bi)$_1$	
(0)L(Beta-Mg_3Bi_2, Mg: Bi: Bi) = -210083-114*T+6*G_{Mg}^{Ser}+4*G_{Bi}^{Ser}	[53]
(0)L(Beta-Mg_3Bi_2, Mg: Va: Bi) = +43728+88*T+6*G_{Mg}^{Ser}+1*G_{Bi}^{Ser}	[53]
(0)L(Beta-Mg_3Bi_2, Mg: Bi, Va: Bi) = -173000-20*T	[53]
(1)L(Beta-Mg_3Bi_2, Mg: Bi, Va: Bi) = -5000	[53]
Mg_2Ni: (Mg)$_{0.6667}$(Ni)$_{0.3333}$	
(0)L(Mg_2Ni, Mg: Ni) = -20320.4+138.49311*T-24.9354*T*ln(T)-1.538E-3*T**2+133805*T**(-1)	[46]

$MgNi_2$: $(Mg, Ni)_{0.3333}(Mg, Ni)_{0.6667}$	
(0)L($MgNi_2$, Mg: Ni) = -24688.46+147.27013*T-25.7998*T*ln(T)-2.46496E-3*T**2+111575.5*T**(-1)	[46]
(0)L($MgNi_2$, Mg: Mg) = +764.7058861 + 20.5882353*T+1*G_{Mg}^{Ser}	[46]
(0)L($MgNi_2$, Ni: Mg) = +1000+0.3333*G_{Ni}^{Ser}+0.6667*G_{Mg}^{Ser}	[46]
(0)L($MgNi_2$, Ni: Ni) = +5254.902+6.862745*T+1*G_{Ni}^{Ser}	[46]
Bi_3Ni: $(Bi)_{0.75}(Ni)_{0.25}$	
(0)L(Bi_3Ni, Bi: Ni) = -5393+0.75*G_{Bi}^{Ser}+0.25*G_{Ni}^{Ser}	[54]
BiNi: $(Bi)_{0.3334}(Ni)_{0.3333}(Bi, Va)_{0.3334}$	
(0)L(BiNi, Bi: Ni: Bi) = 0.6667*G_{Bi}^{Ser}+0.3333*G_{Ni}^{Ser}	[54]
(0)L(BiNi, Bi: Ni: Va) = -3550+0.3334*G_{Bi}^{Ser}+0.3333*G_{Ni}^{Ser}	[54]
(0)L(BiNi, Bi: Ni: Bi, Va) = -1647+1.434*T	[54]
MgNiBi: $(Mg)_1(Ni)_1(Bi)_1$	
(0)L(MgNiBi, Mg: Ni: Bi) = -107000+2.5*T+G_{Mg}^{Ser}+G_{Ni}^{Ser}+G_{Bi}^{Ser}	[*]

[∗]-this work

Supplementary Table 7| Parameters for calculation of phase equilibria in Mg-Sb-Fe system.

Thermodynamic parameters	Ref.
Liquid: (Mg, Fe, Sb, Mg_3Sb_2)$_1$	
(0)L(Liquid, Mg, Sb) = -64574.5-13.8*T	[48]
(1)L(Liquid, Mg, Sb) = -1*T	[48]
(0)L(Liquid, Mg_3Sb_2) = -261367+125.8*T-9.85*T*ln(T)+3*G_{Mg}^{Liq}+2*G_{Sb}^{Liq}	[48]
(0)L(Liquid, Mg, Mg_3Sb_2) = -14941.2-14.3663*T	[48]
(1)L(Liquid, Mg, Mg_3Sb_2) = +10399.95+3*T	[48]
(0)L(Liquid, Mg_3Sb_2, Sb) = -19774.6-21.997*T	[48]
(1)L(Liquid, Mg_3Sb_2, Sb) = -17802.3+3.3426*T	[48]
(0)L(Liquid, Fe, Sb) = -22891.21+17.47*T	[57]
(1)L(Liquid, Fe, Sb) = -15222.54+8.74*T	[57]
(2)L(Liquid, Fe, Sb) = +2336.64	[57]
(0)L(Liquid, Fe, Mg) = +61343+1.5*T	[56]
(1)L(Liquid, Fe, Mg) = -2700	[56]
(0)L(Liquid, Mg, Fe, Mg_3Sb_2, Sb) = -20000	*
Fcc_A1: (Mg, Fe, Sb)$_1$(Va)$_1$	
(0)L(Fcc_A1, Fe, Mg: Va) = 65200	[56]
(0)L(Fcc_A1, Fe, Sb: Va) = +2684.10+21.15*T	[57]
(1)L(Fcc_A1, Fe, Sb: Va) = -21095.63	[57]
(0)L(Fcc_A1, Mg, Ni, Sb: Va) = +500000	*
Bcc_A2: (Mg, Fe, Sb)$_1$(Va)$_3$	
(0)L(Bcc_A2, Fe, Mg: Va) = 66700	[56]
(0)L(Bcc_A2, Fe, Sb: Va) = +4764.10+26.28*T	[57]
(1)L(Bcc_A2, Fe, Sb: Va) = -35112.52	[57]
(0)L(Bcc_A2, Mg, Sb: Va) = +80000	[*]
(0)L(Bcc_A2, Fe, Mg , Sb: Va) = +80000	[*]
Hcp_A3: (Mg, Fe, Sb)$_1$(Va)$_{0.5}$	[56]
(0)L(Hcp_A3, Fe, Mg: Va) = +92400	[57]
(0)L(Hcp_A3, Mg, Sb: Va) = -61998.6-18.999*T	[48]
(0)L(Hcp_A3, Fe, Sb: Va) = +5000	[57]
(0)L(Hcp_A3, Mg, Fe, Sb: Va) = +500000	[*]
Alpha-Mg_3Sb_2: (Mg)$_3$(Sb, Va)$_2$	
(0)L(Alpha-Mg_3Sb_2, Mg: Sb) = -294509-14.05*T+11.212*T*ln(T)-0.013*T**2+3*G_{Mg}^{Ser} +2*G_{Sb}^{Ser}	[48]
(0)L(Alpha-Mg_3Sb_2, Mg: Va) = +40665.1+9*T+3*G_{Mg}^{Ser}	[48]
(0)L(Alpha-Mg_3Sb_2, Mg: Sb, Va) = -13675.1+5.432*T	[48]
(1)L(Alpha-Mg_3Sb_2, Mg: Sb, Va) = -14000+7.476*T	[48]

Beta- Mg_3Sb_2: $(Mg)_6(Sb, Va)_3(Sb)_1$	[48]
(0)L(Beta-Mg_3Sb_2, Mg: Sb: Sb) = -386039-190.64*T+17.16*T*ln(T)+6*G_{Mg}^{Ser}+4*G_{Sb}^{Ser}	[48]
(0)L(Beta-Mg_3Sb_2, Mg: Va: Sb) = -33000.4-15.1*T+14.25*T*ln(T)+6*G_{Mg}^{Ser}+1*G_{Sb}^{Ser}	[48]
(0)L(Beta-Mg_3Sb_2, Mg: Sb, Va: Sb) = -68212.8-29.878*T	[48]
(1)L(Beta-Mg_3Sb_2, Mg: Sb, Va: Sb) = -106813-28.0*T	[48]
FeSb: $(Fe)_1(Fe, Sb)_1$	
(0)L(FeSb, Fe:Sb) = -32715.38+7.00*T +G_{Fe}^{Ser}+G_{Sb}^{Ser}	[57]
(0)L(FeSb, Fe:Fe, Sb) = +16407.28+4.39*T	[57]
(1)L(FeSb, Fe:Fe, Sb) = +26981.65	[57]
$FeSb_2$: (Fe)1(Sb)2	
(0)L($FeSb_2$, Fe:Sb) = -30186.21+9.15*T +G_{Fe}^{Ser}+G_{Sb}^{Ser}	[57]
$Mg_{14}FeSb$: $(Mg)_{14}(Fe)_1(Sb)_1$	
(0)L($Mg_{14}FeSb$, Mg:Fe:Sb) = +181000+19.11*T +G_{Fe}^{Ser}+14*$G_{Mg}^{Ser} + G_{Sb}^{Ser}$	[*]
Mg_6FeSb: $(Mg)_6(Fe)_1(Sb)_1$	
(0)L(Mg_6FeSb, Mg:Fe:Sb) = +12000+11.47*T +G_{Fe}^{Ser}+6*$G_{Mg}^{Ser} + G_{Sb}^{Ser}$	[*]

[∗]-this work

Supplementary Table 8| Parameters for calculation of phase equilibria in Pb-Te-Fe system.

Thermodynamic parameters	Ref.
Liquid: (Pb,Fe,Te,PbTe,Fe_2Te_2)$_1$	
(0)L(Liquid, Fe, Pb) = 110114.85-9.1142*T	[59]
(1)L(Liquid, Fe, Pb) = 27699.55-6.7433*T	[59]
(0)L(Liquid, Fe_2Te_2) = -67060+10*T+2*G_{Fe}^{Liq}+2*G_{Te}^{Liq}	[60]
(0)L(Liquid, Fe_2Te_2, Te) = -11094	[60]
(1)L(Liquid, Fe_2Te_2, Te) = +690	[60]
(2)L(Liquid, Fe_2Te_2, Te) = +7688	[60]
(0)L(Liquid, PbTe) =-61700+20.87*T+G_{Pb}^{Liq}+G_{Te}^{Liq}	[58]
(0)L(Liquid, Pb, PbTe) = +15965.83-3.8*T	[58]
(1)L(Liquid, Pb, PbTe) =+3681.91	[58]
(0)L(Liquid, PbTe, Te) =-6216.19+5.56*T	[58]
(1)L(Liquid, PbTe, Te) = +1174.92	[58]
(0)L(Liquid, Pb, Fe, Te) = -146000	[*]
Fcc_A1: (Fe, Pb, Te)$_1$(Va)$_1$	
(0)L(Fcc_A1, Te:Va) = +35700+G_{Te}^{Ser}	[60]
(0)L(Fcc_A1, Fe, Pb:Va) = 7.0644+90.0518*T	[59]
(0)L(Fcc_A1, Pb, Fe, Te:Va) = +500000	[*]
Bcc_A2: (Fe, Pb, Te)$_1$(Va)$_3$	
(0)L(Bcc_A2, Fe, Pb:Va) = 108.2473+88.9995*T	[59]
(0)L(Bcc_A2, Fe, Te:Va) = -22594	[60]
(0)L(Bcc_A2, Pb, Fe, Te:Va) = +500000	[*]
PbTe: (Pb, Te,Va)$_1$(Pb, Te,Va)$_1$	
(0)L(PbTe, Pb: Te) = 5.458*T-65055.475 + G_{Pb}^{Ser}+G_{Te}^{Ser}	[60]
(0)L(PbTe, Pb: Pb) = 174091.2 +2*G_{Pb}^{Ser}	[60]
(0)L(PbTe, Te: Te) = -157960.355+2*G_{Te}^{Ser}	[60]
(0)L(PbTe, Pb, Te:Va) = -86805.405-32.657*T	[60]
(0)L(PbTe, Va : Pb, Te) =0	[60]
Beta_1: (Fe, Va)$_3$(Te)$_2$	[60]
(0)L(Beta-1, Pb: Te) = -30174-31.442*T+3*G_{Fe}^{Ser}+2*G_{Te}^{Ser}	[60]
(0)L(Beta-1, Va: Te) = 10000+2*G_{Te}^{Ser}	[60]
(0)L(Beta-1, Fe, Va: Te) = -70216	[60]
(1)L(Beta-1, Fe, Va: Te) = -20305	[60]
Beta: (Fe)$_1$(Te)$_1$(Fe, Va)$_1$	
(0)L(Beta, Fe: Te: Fe) = -30715+2*G_{Fe}^{Ser}+G_{Te}^{Ser}	[60]
(0)L(Beta, Fe: Te: Va) = -30174+230.496*T-46.536*T*ln(T)-0.01223*T**2+ 43100*T**(-1)	[60]

(0)L(Beta, Fe: Te: Fe, Va) = -118966+19.997*T	[60]
(1)L(Beta, Fe: Te: Fe, Va) = -46154	[60]
Sigma/Sigma-1: $(Fe, Va)_1(Fe, Va)_1(Te)_2$	
(0)L(Sigma/Sigma-1, Fe: Fe: Te) = -15417-47.631*T+2*G_{Fe}^{Ser}+2*G_{Te}^{Ser}	[60]
(0)L(Sigma/Sigma-1, Va: Fe: Te) = -30222-21.517*T+G_{Fe}^{Ser}+2*G_{Te}^{Ser}	[60]
(0)L(Sigma/Sigma-1, Fe: Va: Te) = -30222-21.517*T+G_{Fe}^{Ser}+2*G_{Te}^{Ser}	[60]
(0)L(Sigma/Sigma-1, Va: Va: Te) = +13547+2*G_{Te}^{Ser}	[60]
(0)L(Sigma/Sigma-1, Fe, Va: Fe: Te) = -55623+48.634*T	[60]
(1)L(Sigma/Sigma-1, Fe, Va: Fe: Te) = -3328	[60]
(0)L(Sigma/Sigma-1, Fe: Fe, Va: Te) = -56003+48.551*T	[60]
(1)L(Sigma/Sigma-1, Fe: Fe, Va: Te) = -5291	[60]
Yita: $(Fe, Va)_1(Te)_2$	
(0) L(Yita, Fe: Te) = -73469+318.524*T-63.9656*T*LN(T)-0.020402*T**2 +107200*T**(-1)	[60]
(0)L(Yita, Va: Te) = +10700+2*G_{Te}^{Ser}	[60]
(0)L(Yita:, Fe, Va: Te) = +62432-71.949*T	[60]
Gama: $(Fe)_1(Te)_{1.183}$	
(0)L(Gama, Fe:Te) = -23492-17.005*T+G_{Fe}^{Ser}+1.183*G_{Te}^{Ser}	[60]

[∗]-this work

Supplementary Table 9| Estimated uncertainty of the ΔT_{Cu}, measured heat flow (Q), and conversion efficiency (η) of the Zintl-based module in this study.

ΔT (K)	ΔT_{Cu} (K)	$\delta(\Delta T_{Cu})$ (%)	$\delta(Q)$ (%)	$\delta(\eta)$ (%)
30	0.04	32.06	32.85	32.55
80	0.17	8.41	11.02	10.88
130	0.29	4.80	8.60	8.42
180	0.41	3.42	7.90	7.65
230	0.52	2.72	7.63	7.29
280	0.65	2.18	7.45	7.04
330	0.77	1.84	7.36	6.86
380	0.90	1.56	7.30	6.72
430	1.03	1.37	7.26	6.62
